# Impact of Heterogeneous 5G Duplex Arrangements on Future Non-Terrestrial IoT Networks

**DOI:** 10.3390/s26196108

**Published:** 2026-09-26

**Authors:** Karina Turzhanova, Alexander Pastukh, Valery Tikhvinskiy, Olga Mironova, Daria Kalugina, Yelizaveta Vitulyova, Olga Abramkina, Farida Abdoldina, Alexey Terekhov, Gleb Tokin

**Affiliations:** 1 Almaty University of Power Engineering and Telecommunications Named After Gumarbek Daukeev, Almaty 050013, Kazakhstan; karinaturzhanova@gmail.com (K.T.); olga.manank@gmail.com (O.A.); 2 The M.I. Krivosheev National Research Centre for Telecommunication (NRCT), 105064 Moscow, Russia; mironovaol@nic-t.ru (O.M.); kaluginadi@nic-t.ru (D.K.); 3 National Research University Higher School of Economics (HSE University), 101000 Moscow, Russia; vtkhvinskiy@gmail.com; 4 International Engineering and Technological University, Almaty 050060, Kazakhstan; 5 Almaty Management University, Almaty 050060, Kazakhstan; 6 Institute of Digital Engineering and Technology, Satbayev University, Almaty 050043, Kazakhstan; 7 National Scientific Laboratory for the Collective Use of Information and Space Technologies (NSLC IST), Satbayev University, Almaty 050043, Kazakhstan; 8 Institute of Automation and Information Technologies, Satbayev University, Almaty 050043, Kazakhstan; abdoldinafarida@gmail.com (F.A.); aterekhov1@yandex.ru (A.T.); 9 Institute of Military Affairs, Satbayev University (KazNRTU), Almaty 050013, Kazakhstan; tokingleb@gmail.com

**Keywords:** Satellite IoT, NTN, D2C, 5G/6G IoT, IMT, MSS, sharing studies

## Abstract

Satellite direct-to-cell connectivity can extend 5G Internet of Things (IoT) services into areas without terrestrial coverage by reusing terrestrial mobile-service spectrum. This study examines aggregate interference at a non-terrestrial network (NTN) satellite receiver in selected bands within 694–2690 MHz, where terrestrial duplex arrangements differ between regions. A satellite receiving an uplink may also receive terrestrial downlink emissions from another country using TDD or an overlapping FDD downlink. Six geographical case studies combine satellite motion, population-based terrestrial deployments, and aggregate-interference calculations over a 180 kHz receiver bandwidth. Results are presented as interference-to-noise ratio (I/N) complementary cumulative distribution functions and geographical maps, with nadir and random off-nadir pointing cases. In the evaluated TDD cases, BS contributions dominate the upper part of the aggregate-interference distributions, and elevated interference extends beyond the emitting countries. These findings identify cross-border interference pathways under the assumed deployments, loading, spectral coupling, and antenna patterns. They do not establish that duplex arrangement outweighs deployment density independently of these assumptions. The adopted −6 dB I/N threshold is a screening reference; service availability, link performance, and the effectiveness of mitigation techniques are not evaluated.

## 1. Introduction

The integration of Internet of Things (IoT) technologies based on non-terrestrial networks (NTNs) into the future 5G/6G ecosystem requires access to scarce spectrum resources [1]. 3GPP has standardized several frequency bands for 5G NTN operation [2]. However, the bands currently standardized for NTNs are already used by global mobile-satellite service (MSS) systems, such as Iridium, Globalstar, and Inmarsat [3]. Although these systems can support IoT applications, deploying a new system in the same bands may be challenging because of the need to ensure compatibility with existing systems [4].

One way to overcome this spectrum shortage is to use frequency bands allocated to the mobile service. Several companies, including AST SpaceMobile, SpaceX, and Lynk [5,6,7], are currently launching satellites and testing direct-to-cell (D2C) services that allow unmodified smartphones to operate in mobile operators’ frequency bands in areas where terrestrial coverage is unavailable. In the IoT sector, OQ Technologies has recently tested 5G IoT services in frequency bands licensed to mobile operators after being granted an experimental license for these trials [8].

NTN connectivity is particularly important for IoT applications such as asset tracking, agriculture, environmental monitoring, utilities [9], logistics, maritime operations, and remote infrastructure. These applications often require wide-area service in locations where continuous terrestrial coverage is unavailable or uneconomic [10], and many rely on small, power-constrained terminals that must operate unattended for long periods [11]. Extending cellular IoT connectivity through satellites can provide service continuity across terrestrial coverage gaps while retaining alignment with the wider mobile-device and operator ecosystem.

For the NTN IoT industry, securing access to suitable terrestrial mobile-service bands is therefore important for both scale and technical performance. Reuse of widely supported sub-3 GHz cellular bands can reduce dependence on separate satellite-specific device variants and can facilitate integration with terrestrial operator networks. However, regulatory access alone does not make a band operationally suitable: the satellite uplink must remain sufficiently protected from aggregate terrestrial emissions across the receiver footprint, including emissions originating in neighboring countries that use a different duplex arrangement.

Within the Radiocommunication Sector of the International Telecommunication Union (ITU-R), the use of mobile service bands in the 694–2690 MHz frequency range for direct connectivity between satellites and International Mobile Telecommunications (IMT) user equipment is being studied during the study cycle leading up to the 2027 World Radiocommunication Conference (WRC-27) [12,13]. The ITU-R is studying and developing the conditions under which D2C services could operate in these bands, focusing primarily on interference from satellite systems into terrestrial cellular networks. At the same time, the reverse interference scenario—from terrestrial systems into D2C systems—has received comparatively limited attention in the literature that was reviewed here. In such a scenario, the satellite receiver is likely to be the most exposed component because of its wide coverage area and the resulting aggregate interference received from large territories in which terrestrial systems are deployed.

Aggregate terrestrial interference is therefore relevant to the selection of a D2C frequency band and service region. Incomplete information about terrestrial deployments limits the accuracy of such assessments. The present study examines selected interference scenarios to identify where more detailed, system-specific assessment may be needed.

This article compares terrestrial interference in selected frequency bands and geographical areas to inform interference screening for NTN IoT band selection.

The study addresses three questions: (i) how BS and UE contributions differ in the selected interference scenarios; (ii) whether elevated interference extends beyond the countries containing the modeled transmitters; and (iii) how the reported patterns depend on satellite pointing and the stated deployment assumptions. The six cases differ in geography, frequency, and terrestrial deployment. They are not a controlled experiment in which duplex configuration alone is varied, and they cannot establish a causal ranking of duplex arrangement and deployment density.

The contribution is an application-specific comparison of terrestrial-to-NTN uplink interference under cross-border frequency-direction overlaps in sub-3 GHz mobile bands. Established aggregate-interference and ITU-R modeling approaches are used rather than a new propagation or Monte Carlo method. The analysis connects regional duplex arrangements to the source of interference received by a moving satellite and distinguishes BS and UE contributions within the evaluated TDD cases. It shows why a review of terrestrial uplink use in the intended service country alone can miss a downlink interference pathway from another country. The resulting insights remain conditional on the modeled deployments and receiver geometry.

## 2. Parameters and Assumptions

Two frequency-direction relationships are considered. When terrestrial and NTN systems use aligned FDD uplink and downlink directions, the satellite receive channel overlaps terrestrial UE uplink transmissions. In the corresponding cases modeled here, interference is therefore aggregated from UEs.

When the NTN uplink overlaps a terrestrial FDD downlink or a TDD band, terrestrial BS transmissions can also enter the satellite receive channel. In a TDD network, BS downlink and UE uplink transmissions occupy different transmission intervals within a cell [14,15,16]. Their contribution to a satellite receiver depends on the DL/UL allocation, loading, synchronization, and the satellite’s visibility of the transmitting networks.

Figure 1 below illustrates the two interference scenarios: one in which the NTN satellite is exposed only to interference from UEs and another in which it is exposed to interference from both BSs and UEs.

Comprehensive operator BS inventories were not available for this study. Terrestrial deployment densities were therefore inferred using the Ra/Rb approach applied in ITU-R WP 5D sharing studies [17,18], followed by redistribution using population data. This produces a modeled deployment rather than a measured inventory. The regional density is expressed as follows:

$${Dl}_{urban/suburban}={Ds}_{urban/suburban}*{Ra}_{urban/suburban}*Rb$$

where Ds is the density of simultaneously active BS transmitters per km^2^ for the reviewed coverage area; this value is provided in Table 1; Ra (%) is a ratio of coverage areas to areas of cities/built areas/districts; Rb (%) is a ratio of built areas to the total area of the region under study. Table 1 provides the values for Ra and Rb coefficients.

For the TDD cases, let N denote the modeled sector population obtained from the deployment-density calculation using Ra and Rb, with the adopted frequency adjustment, before applying TDD activity or network loading. The simultaneously active BS count is N × 0.75 × 0.2. The simultaneously active UE count is N × 0.25 × 0.2 × 3, where 0.25 represents the uplink portion in which the corresponding BS sectors do not transmit downlink, 0.2 is the network loading factor, and 3 is the assumed number of UEs per sector. Both counts therefore start from the same sector population: the UE calculation uses the 0.25 uplink factor instead of the 0.75 downlink factor and does not inherit the downlink reduction. The resulting active BS and UE counts enter the aggregate-interference sums; these count factors are not applied again to the summed contributions. No additional 50% average BS activity factor was used. The modeled BSs were distributed spatially using population data. The 6 GHz deployment approach was adapted to the studied sub-3 GHz bands using an approximate density normalization. The cited free-space propagation comparison between 6 GHz and 2.6 GHz gives a difference of approximately 7.3 dB [19]; the adopted divisor was $F_{adjust}=\sqrt{10^{7.3/10}}=2.317$.

The divisor is a scenario assumption, not a validated law relating frequency to BS density. A free-space path-loss difference alone does not determine a cell’s coverage area or the number of sites required: terrain, clutter, antenna height, coverage targets, and capacity demand also matter. Using a single normalization across 694–2690 MHz therefore introduces uncertainty, especially away from the 2.6 GHz reference comparison. No density-factor sweep is reported, so the geographical extent of threshold exceedance and the ordering of scenarios are not claimed to be robust to alternative deployment assumptions.

Parameters of the terrestrial networks can be found in the Report ITU-R M.2292 [20], Recommendation ITU-R M.2101 [21] and WP 5D documents [22]. Parameters used in the simulations are presented in Table 2 below:

Table 3 below shows the frequency bands where interference from terrestrial networks to the NTN satellite receivers is possible within the 694–2690 MHz frequency range. The table also specifies in which bands BS interference is possible and in which UE-only interference can occur.

As can be seen from the above table, interference from terrestrial base stations may occur in the S band because of the extensive use of TDD in this frequency range, as well as in UHF bands where terrestrial and NTN systems may use opposite transmission directions. This study considers several representative frequency bands and geographical areas in which the satellite receiver may be exposed either to interference from UEs only or to interference from terrestrial BSs.

For the S-band BS-interference case, the study considers China and Japan with terrestrial TDD operation in n41 and an NTN receiver in the overlapping n7 uplink. The cited deployment information for China is given in [23]. The UHF case considers the overlap between the n14 uplink in the United States and the n28 downlink in Mexico [24]. The UE-only cases include Australia in n7 and Europe in n1, where the modeled terrestrial and NTN uplink directions are aligned [25]. Section 4.5 and Section 4.6 additionally examine the United States in n26 and interference from US n41 deployments into surrounding n7 regions.

It should be noted that when calculating BS density in all scenarios, we have considered Ra2Rb2 for Europe, China/Japan and the United States, and Ra1Rb1 for other regions; this is due to the fact that the first 3 regions have a high density of base stations and high population, which requires denser networks to provide sufficient capacity.

Table 4 lists the satellite parameters adopted for these scenarios, based on material submitted to WP 4C for WRC-27 agenda item 1.13 [26]. They describe the modeled receivers and do not represent all NTN systems or constellations.

For each 180 kHz victim channel, receiver thermal noise was evaluated as N = kTB, where k is Boltzmann’s constant, T is the system noise temperature in Table 4, and B is the receiver noise bandwidth. Interference contributions were aggregated in linear power units before I/N was expressed in decibels. The study adopts −6 dB as a common I/N screening reference, following the IMT-Advanced protection criterion in Report ITU-R M.2292 [20]. Its use here is a study assumption, not an NTN-specific regulatory determination. Exceeding this reference identifies increased interference relative to receiver noise; it does not, by itself, establish outage, packet error rate, throughput, or link availability. Likewise, a value below the reference does not establish service availability without a desired-signal link budget. An alternative reference can be read against the same CCDF; it changes the exceedance classification, not the underlying I/N samples. Geographical percentages under alternative thresholds are not evaluated here.

Figure 2 and Figure 3 show the satellite receive-antenna patterns used in the simulations. The construction adapts the circular Taylor-illumination formulation discussed in Section 1.4 of Recommendation ITU-R S.1528 [27] to a rectangular effective aperture using the separable principal-plane model below. It is an assumed receive pattern rather than a validated model of a particular flight antenna. Input characteristics of the antenna pattern from Recommendation ITU-R S.1528 are provided in Table 5 below.

The Taylor circular-illumination aperture function can be described as follows [28]:

$$F\left(u\right)=\frac{2J_{1}\left(u\right)}{u}\prod_{i=1}^{l-1}\frac{N_{i}\left(u\right)}{D_{i}\left(u\right)},\;\;\;F\left(0\right)=1$$

where

$$N_{i}\left(u\right)=1-\frac{u^{2}}{\pi^{2}\sigma^{2}\left[A^{2}+{\left(i-\frac{1}{2}\right)}^{2}\right]},\;D_{i}\left(u\right)=1-{\left(\frac{u}{\pi u_{i}}\right)}^{2}$$

With the corresponding gain:

$$G\left(u\right)=G_{max}+20log\left|F(u)\right|\;\;[\mathrm{dBi}]$$

The three shape parameters follow from SLR and l alone:

$$A=\frac{arcosh\left(10^{SLR/20}\right)}{\pi },\;\;\;\;\;u_{i}=\frac{j_{1,\;i}}{\pi },\;\;\;\;\;\sigma =\frac{u_{1}}{\sqrt{A^{2}+{\left(l-\frac{1}{2}\right)}^{2}}}$$

The antenna frame is right-handed with the boresight around the +x; the direction is defined by azimuth, and elevation has the following unit vector:

$$n=\left(cos\;\varepsilon \;cos\;\alpha ,\;cos\;\varepsilon \;sin\;\alpha ,\;sin\;\varepsilon \right)=\left(cos\;\theta ,sin\;\theta \;cos\;\varphi ,\;sin\;\theta \;sin\;\varphi \right)$$

where

$$\theta =\arccos n_{x},\;\;\varphi =atan2\left(n_{z},\;n_{y}\right),\;n_{y}=\sin\theta \cos\varphi ,\;\;n_{z}=\sin\theta \sin\varphi \;$$

The two aperture arguments are the projections of n onto the main axes of the radiating area as follows:

$$u_{r}=\frac{\pi L_{r}\left|n_{y}\right|}{\lambda }=\frac{\pi L_{r}\left|\sin\theta \cos\varphi \;\right|}{\lambda },\;{\;\;\;\;\;\;\;\;u}_{t}=\frac{\pi L_{t}\left|n_{z}\right|}{\lambda }=\frac{\pi L_{t}\left|\sin\theta \sin\varphi \;\right|}{\lambda }$$

This is written as the direction cosines, not in the raw angles of azimuth and elevation. The two coincide in the principal planes (ε = 0 and α = 0) but differ elsewhere by the factor cos ε; the direction-cosine form is single-valued over the whole sphere, whereas $\left|\sin\alpha \right|$ is degenerate at the ε = ±90 degrees, where a single physical direction would otherwise be assigned a continuum of gain values.

The two principal-plane patterns are treated as independent and multiplied as follows:

$$G\left(\theta ,\;\varphi \right)=G\left(u_{r}\right)+G\left(u_{t}\right)-G_{max}=G_{max}+20log\left|F\left(u_{r}\right)F(u_{t})\right|$$

With F from (1) and the aperture arguments from (7), F(0) = 1 preserves the specified peak gain. The model assumes a rectangular effective radiating area with independent illumination tapers along its principal axes. This produces a rectangular sidelobe structure with ridges along the principal planes and deep minima between them; these features are properties of the adopted pattern.

Figure 2 and Figure 3 illustrate antenna patterns for UHF and S bands that were used in the simulations as the satellite’s receiving antennas.

## 3. Methodology

A hybrid deterministic–Monte Carlo simulation was used to estimate aggregate terrestrial interference at a victim NTN satellite receiver. Satellite motion was propagated deterministically using Keplerian orbital elements with a one-second time step. At every orbital step, a Monte Carlo snapshot of the terrestrial IMT network was generated according to Recommendation ITU-R M.2101 [21]. Interference from the applicable BS and UE transmissions was then evaluated using that terrestrial snapshot and the satellite geometry at the same step. The simulated duration was six months per scenario. Thus, each one-second orbital step corresponds to one terrestrial Monte Carlo snapshot, and the number of generated snapshots equals the number of simulated orbital steps. The resulting I/N statistics combine deterministic orbital evolution with probabilistic terrestrial-network modeling. This outer one-second sampling step does not define the TDD downlink/uplink slot timing.

Observation–duration convergence was assessed by recomputing I/N CCDFs over successive extensions of the simulated interval and comparing them at common exceedance probabilities. Convergence was defined by a maximum horizontal separation of 0.1 dB over the evaluated probability range. This check assesses the stability of the sampled distribution as the simulation interval is extended; it does not provide confidence intervals or quantify sensitivity to alternative deployment assumptions. The one-to-one correspondence between orbital steps and terrestrial snapshots specifies the sampling procedure, rather than establishing the statistical independence of every sampled input.

The BS and UE plots distinguish source contributions under each scenario’s assumptions. A CCDF reports P(X > x), where X is the simulated aggregate I/N in dB and x is the value on the horizontal axis. The vertical scale is logarithmic; the red vertical line indicates the −6 dB screening reference. A 95th or 99th percentile corresponds to an exceedance probability of 0.05 or 0.01, respectively. This defines how to read the existing curves; no additional percentile estimates or uncertainty intervals are reported. The evaluated sample distribution is not automatically a probability for an arbitrary user, geographical area, or service period.

The interference from the *i*-th terrestrial base station was calculated using the following expression [29,30]:

$$I_{BS,\;\;i}=\frac{P_{BS,i}G_{BS,i}(\varphi ,\;\theta )G_{NTN}(\varphi ,\;\theta )}{L_{base}L_{clutter}L_{feeder}}$$

where

$P_{BS,i}$—transmitted power of the base station;

$G_{BS,i}(\varphi ,\;\theta )$—gain of the transmitting BS’s antenna towards the direction of the NTN receiver;

$G_{NTN}(\varphi ,\;\theta )$—gain of the NTN receiver’s antenna towards the interfering BS;

$L_{base}$—free space losses based on Rec. P.619 [19];

$L_{clutter}$—clutter losses for the BS below rooftop according to Rec. P.2108 [31,32];

$L_{feeder}$—feeder losses.

The interference from the *j*-th terrestrial user equipment was calculated using the following expression [29,30]:

$$I_{UE,j}=\frac{P_{UE,j}G_{UE,j}G_{NTN}(\varphi ,\;\theta )}{L_{base}L_{clutter}L_{indoor}L_{body}}$$

where

$P_{UE,j}$—transmitted power of the user equipment, W

$G_{UE,j}$—gain of the transmitting UE’s antenna;

$G_{NTN}(\varphi ,\;\theta )$—gain of the NTN receiver’s antenna towards the interfering UE;

$L_{base}$—free space losses based on Rec. P.619 [19];

$L_{clutter}$—clutter losses for the UE in urban areas according to Rec. P.2108 [31,32];

$L_{body}$—user’s body loss [33];

$L_{indoor}$—building entry losses according to Rec. P.2109 [34] for the UEs located indoors.

The general linear-power aggregation is written as follows [29,30]:

$$I_{agg}=\sum_{i}^{N}I_{BS,\;\;i}+\sum_{j}^{N}I_{UE,j}$$

In the aligned-FDD cases, only the UE contribution is included; in the reversed-FDD case, the modeled co-channel contribution is from BSs. For TDD, the summation limits are the simultaneously active BS and UE counts defined in Section 2. The model uses complementary downlink and uplink activity shares of 0.75 and 0.25, with the same 0.2 network loading factor applied separately to each count. Three UEs are assumed per sector during the uplink portion. The resulting BS and UE contributions are aggregated in linear power units, with the activity weighting already represented in the transmitter counts. This statistical treatment does not assume that the full BS and UE populations transmit simultaneously, or that a BS sector transmits downlink during its uplink portion. It represents aggregate activity without specifying the succession of slots in a synchronized network. The one-second orbital/Monte Carlo sampling interval is not a TDD slot duration. The assumed 75%/25% DL/UL allocation is a study input, and alternative allocations or explicit inter-network synchronization schedules have not been evaluated.

The spectral model assumes co-channel coupling over the 180 kHz victim bandwidth. It does not evaluate transmitter emission masks, adjacent-channel leakage ratio (ACLR), receiver adjacent-channel selectivity (ACS), partial channel overlap, or receiver blocking. For an adjacent or partially overlapping channel, a frequency-dependent coupling calculation would be required. Blocking additionally requires a receiver-specific susceptibility model and cannot generally be represented by a simple overlap factor. The results therefore concern the stated co-channel model rather than hardware-specific adjacent-channel compatibility. The baseline satellite receiver beam points to nadir. In the additional off-nadir case, its pointing direction is randomly changed with a 60-degree scan limit at each simulation step to explore a changing receive geometry. Beam pointing was changing at each simulation step, and the azimuth was random as well. This represents idealized random steering, rather than a traffic-driven beam-hopping schedule or the operation of a particular constellation. Figure 4 illustrates the nadir geometry.

Nadir pointing was adopted as the baseline case. Because the worst-case scan angle depends jointly on slant range, the satellite pattern, BS downtilt or beamforming, and the spatial distribution of interferers, off-nadir sensitivity analysis with random pointing was also evaluated. At the same time, the proper beam configuration will depend on the specific constellation.

## 4. Study Results

The CCDFs and maps describe different summaries of the simulated samples. Map axes identify sub-satellite longitude and latitude, rather than national service-area boundaries; with off-nadir pointing, the sub-satellite position and beam center need not coincide. The map color bars specify the displayed mean statistics. A mean computed in dB is not equivalent to averaging linear power ratios and then expressing the result in dB, so map colors using different conventions should not be compared as a controlled pointing experiment. The CCDFs provide the appropriate accompanying view of the sample distributions. Neither a spatial mean nor a visually colored area establishes a country’s temporal exceedance probability or the percentage of national territory exceeding the reference. No country-level medians or area-exceedance percentages are reported.

### 4.1. Australia: n7 Band

The simulation was performed for the n7 band and included an analysis of interference within the national borders of Australia. Given that the n7 band uses FDD and that the downlink/uplink directionality of terrestrial networks is the same as that of the D2C system operating over Australia, only UEs served by terrestrial networks can cause interference to the satellite receivers. Figure 5 presents the deployment density of terrestrial networks.

Figure 6 and Figure 7 present the nadir and random off-nadir CCDFs and maps for UE interference over Australia in n7. Both distributions include exceedances of the −6 dB reference in their upper tails. The maps show higher interference associated with urban deployment concentrations and lower levels over many rural and remote locations. These spatial patterns identify differences within the modeled domain; they do not establish a national service-availability result or imply that every rural location satisfies the reference.

### 4.2. China and Japan: n7/n41 Bands

This case examines interference from modeled n41 TDD deployments in China and Japan into an NTN receiver in the overlapping n7 uplink. Figure 8 shows the assumed BS distribution. The scenario evaluates aggregate I/N under that deployment rather than the availability of a particular D2C service.

Figure 9 presents the BS, UE, and total I/N distributions for the China/Japan case. The total curve follows the BS contribution closely, while the UE contribution is substantially lower. The upper tail reaches positive I/N values of several tens of decibels. This demonstrates BS dominance within the modeled case; it does not establish how much of the difference from other countries is caused by duplex configuration alone. Such high interference could severely impair uplink reception and may cause an outage if the available link margin is insufficient. The CCDF describes I/N exceedance within the evaluated samples, rather than the probability of service outage.

Figure 10 shows the separate spatial distributions of BS and UE interference for nadir pointing over China and Japan.

Figure 11 compares the nadir and random off-nadir I/N distributions outside China and Japan. The BS contribution closely follows the total curve in both cases. Figure 12 and Figure 13 locate elevated interference at sub-satellite positions in surrounding regions, including parts of Mongolia and Russia, the Korean peninsula, the Himalayan border region, and mainland Southeast Asia. These are descriptions of mapped locations, not a ranking of whole countries. The distributions and maps identify a cross-border BS interference pathway under the assumed geometry; no country-wide median, national area-exceedance percentage, or mitigation performance is inferred from them.

Figure 12 and Figure 13 show the corresponding BS and UE interference maps outside China and Japan. The coordinates identify sub-satellite locations. The maps summarize receiver interference over the sampled geometry and do not delineate a national service-outage area.

### 4.3. Europe: n1 Band

The simulation was performed for the n1 band and included an analysis of interference over the European Union and several non-EU countries, namely Switzerland, Norway, the United Kingdom, Albania, Serbia, and Montenegro. Given that the n1 band uses FDD and that the downlink/uplink directionality of terrestrial networks in Europe conforms to that of the D2C system in the n1 band, only interference from UEs served by terrestrial networks was considered. Figure 14 presents the deployment density of terrestrial networks within the European area considered in the study.

Figure 15 and Figure 16 present the nadir and random off-nadir CCDFs and maps for UE interference in the European n1 case. The distributions include positive upper-tail I/N values. Higher mapped levels occur around extensive terrestrial deployment concentrations, including parts of the United Kingdom, the Netherlands, Belgium, and Germany. Lower values at other sampled locations should be interpreted locally, rather than as evidence that whole countries or all remote areas satisfy the −6 dB reference. The results concern receiver interference under the modeled European deployment.

### 4.4. US-Mexico: n14/n28 Bands

For this geographical area, the analysis considers the reverse duplex arrangements of the n14 and n28 bands. In Mexico, the n28 band is used for terrestrial networks, whereas the n14 band is used in the United States. Therefore, if D2C IoT is deployed over the United States in the n14 band, the satellite receiver may be subject to interference from base stations deployed in Mexico. Figure 17 presents the deployment of base stations in Mexico.

Figure 18 and Figure 19 show interference from Mexican BSs into the n14 satellite receive channel for sub-satellite positions over the United States. In the nadir map, elevated values are concentrated toward the southern United States, with the strongest mapped interference near Mexico. The random off-nadir case changes the spatial pattern, so the nadir interpretation cannot be extended to all pointing geometries. The corresponding CCDFs describe the distribution of I/N under each case. Neither map establishes the availability of an n14 D2C uplink, and meeting the reference in part of the nadir map does not establish that the rest of the United States is protected under arbitrary steering.

### 4.5. USA: n26 Band

This case examines terrestrial UE interference in the n26 uplink over the United States. The modeled terrestrial and NTN systems have aligned FDD directions, so only UE emissions enter the satellite receive channel in this scenario. Figure 20 shows the deployment distribution used to model the terrestrial network.

Figure 21 and Figure 22 present the nadir and random off-nadir UE-interference CCDFs and maps over the United States. Higher interference is associated with urban deployment concentrations, while many rural and remote locations have lower levels. The CCDFs nevertheless contain an upper tail above −6 dB. Lower interference at rural or remote locations does not establish that every such location meets the reference. Satisfaction of that reference must be assessed at the relevant I/N value and geometry, and no link-outage or service-availability conclusion follows from these plots alone.

### 4.6. USA: n7/n41 Bands

This scenario considers the overlap between the n7 and n41 bands in North and South America. In the United States, the n41 band is used by terrestrial networks in TDD mode, whereas Mexico, Brazil, Colombia, and several other South American countries use the FDD n7 band. Consequently, a satellite serving areas in Mexico and South America in the n7 band may be subject to interference from base stations deployed in the United States. Figure 23 presents the deployment of base stations within the United States considered in the simulation.

Figure 24 presents the BS, UE, and total I/N CCDFs outside the United States for nadir and random off-nadir pointing. Figure 25 and Figure 26 show the corresponding spatial distributions. In both CCDFs, the BS curve closely follows the total, while the UE contribution is lower. The maps show elevated BS interference at sub-satellite positions over Mexico and parts of Central America and the Caribbean, extending toward the northern part of South America. The strength and extent of the pattern depend on pointing and on the map statistic. These observations describe the plotted receiver geometries; they do not quantify the percentage of any country’s territory exceeding −6 dB or establish national service availability.

## 5. Discussion

The six cases illustrate how terrestrial transmissions can enter an NTN IoT satellite receive channel under different regional frequency arrangements. Within the evaluated TDD cases, the BS component closely follows the total I/N CCDF, while the UE component is lower. The aligned-FDD cases also show substantial spatial variation in UE interference. These results identify interference sources and geographical patterns conditional on the modeled inputs. Because the cases differ in deployment, frequency, and geometry, the comparison does not isolate duplex mismatch as the primary cause independently of density.

High positive I/N, for example +10 or +15 dB, indicates interference substantially above receiver thermal noise and can severely impair a power-limited NTN uplink. Outage is a plausible consequence if the resulting signal-to-interference-plus-noise ratio falls below the receiver’s decoding requirement. Its likelihood cannot be inferred from I/N alone because it also depends on desired-signal strength, waveform and coding, and receiver processing. Other potential IoT consequences include additional access attempts or repetitions, increased latency, reduced capacity, and greater terminal energy use. These effects are not quantified here; the study does not report an outage probability, packet error rate, throughput, or terminal energy result.

For Australia in n7, Europe in n1, and the United States in n26, the modeled receiver is exposed to UE uplink emissions. Their CCDFs show that −6 dB exceedances are concentrated farther into the upper tail than in the BS-inclusive TDD cases. The spatial maps show lower interference at many locations away from dense terrestrial deployments. These observations support location-specific screening, rather than a conclusion that aligned FDD guarantees compatibility or that all rural areas meet the chosen reference.

The China/Japan and US n7/n41 cases show BS contributions that dominate the total CCDFs under the assumed TDD treatment. The corresponding maps indicate that receiver interference can extend beyond the emitting countries. The reversed-FDD US–Mexico case provides another route by which a terrestrial downlink overlaps an NTN uplink. Random off-nadir pointing changes the sampled antenna coupling and spatial distribution, so geographical statements from a nadir map should not be generalized to arbitrary steering.

Duplex arrangement is therefore relevant when identifying potential interference sources for NTN IoT band selection. An overlap with terrestrial downlink spectrum introduces BS emissions into the satellite receive channel, whereas the aligned-FDD cases considered here contain UE emissions. The magnitude of aggregate I/N also depends on transmitter density and placement, radiated power, loading, propagation, spectral coupling, and satellite gain toward those transmitters. The study does not establish a universal ranking of these factors.

The selected cross-border cases illustrate specific overlapping frequency directions. They are not a survey of the prevalence of such arrangements, of national coordination practices, or of the causes of differences between national frequency plans. Applying the findings to another region requires checking the bands and transmission directions actually deployed there.

Cross-border terrestrial deployments should be included in an NTN interference assessment whenever they are visible to the satellite and overlap its receive channel. Candidate responses include beam selection, improved spatial discrimination or nulling, scheduling, and interference suppression. Their effectiveness is not evaluated here, and no suppression gain or recovered service area is claimed. A useful next study would compare a specified mitigation method with the same unmitigated deployment and receiver assumptions, including its effect on the desired uplink.

## 6. Conclusions

This study compares aggregate terrestrial interference at an NTN IoT receiver across six geographical cases in sub-3 GHz mobile bands. The results show BS-dominated interference in the evaluated TDD cases and cross-border interference where terrestrial downlink transmissions overlap an NTN uplink. These are conditional scenario findings. The analysis does not separate the causal effect of duplex configuration from differences in deployment density, frequency, and geometry.

The BS-inclusive cases contain high positive I/N values, including at sub-satellite positions outside the emitting countries. The CCDFs describe the exceedance distribution within the simulated domains, while the maps locate spatial variation. Neither provides a country-wide outage probability or the fraction of national territory losing service.

The aligned-FDD cases expose the satellite receiver to UE emissions and generally show lower interference than the BS-inclusive cases, with higher values around dense deployment concentrations. Exceedances of the −6 dB screening reference remain possible. Aligned duplex direction is therefore useful when screening candidate bands, but it is not sufficient to establish interference protection or D2C service availability.

Band selection should account for actual terrestrial frequency directions in the intended service region and in other regions visible to the receiver. The nadir and random off-nadir cases also show why pointing assumptions must accompany geographical interpretations. Candidate mitigation measures require separate evaluation; their benefit cannot be inferred from an I/N exceedance alone.

The results identify cases requiring more detailed system-specific assessment. Further work should validate deployments against operator or measured data, examine density and loading sensitivity, specify realistic TDD scheduling and satellite beam steering, and evaluate receiver selectivity and practical mitigation with a desired-signal link budget.

Deployment densities are inferred from Ra/Rb factors and population redistribution rather than measured operator inventories. The 6 GHz frequency scaling is approximate, and no density-factor sensitivity study is reported. Temporal activity is idealized, and random off-nadir pointing is not a constellation-specific beam schedule. Hardware-specific selectivity, blocking, polarization mismatch, and nonlinear effects are not modeled. The study also does not provide country-level area statistics or uncertainty intervals across independent deployment realizations. Consequently, the results do not establish robustness to these assumptions, definitive coordination distances, regulatory approval, or commercial service availability.

## Figures and Tables

**Figure 1:**
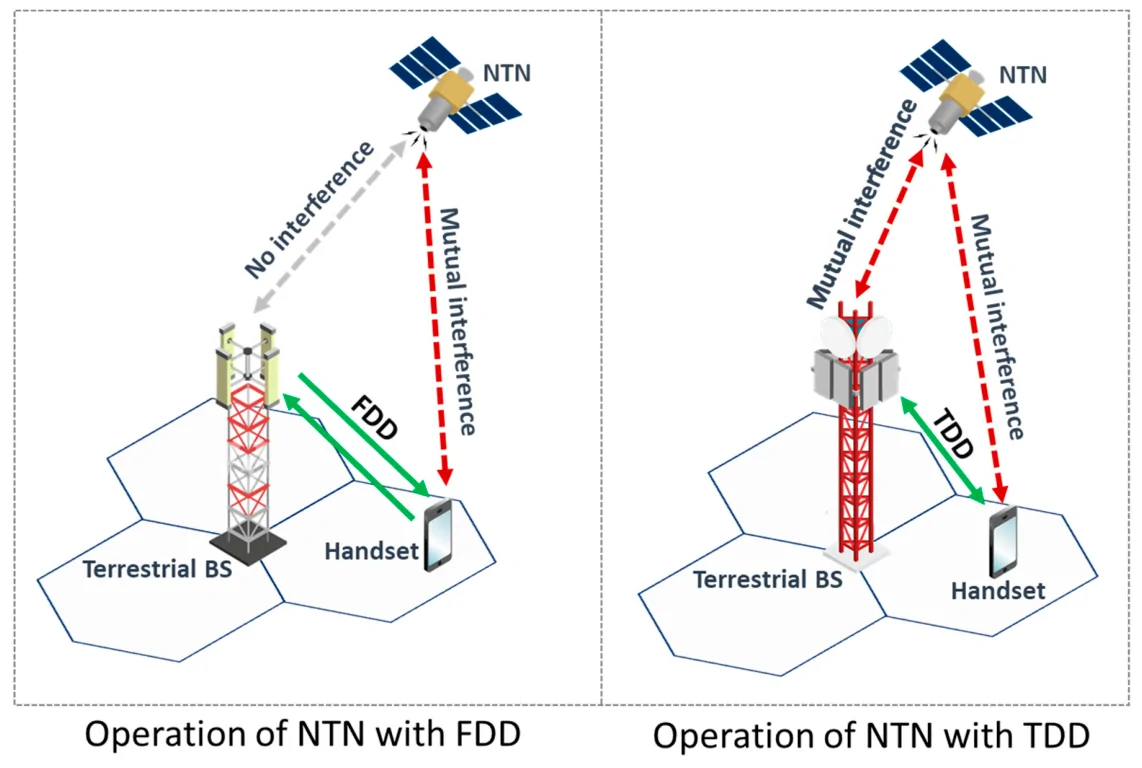
Interference from terrestrial IMT networks to the NTN receiver for different operational duplexes.

**Figure 2:**
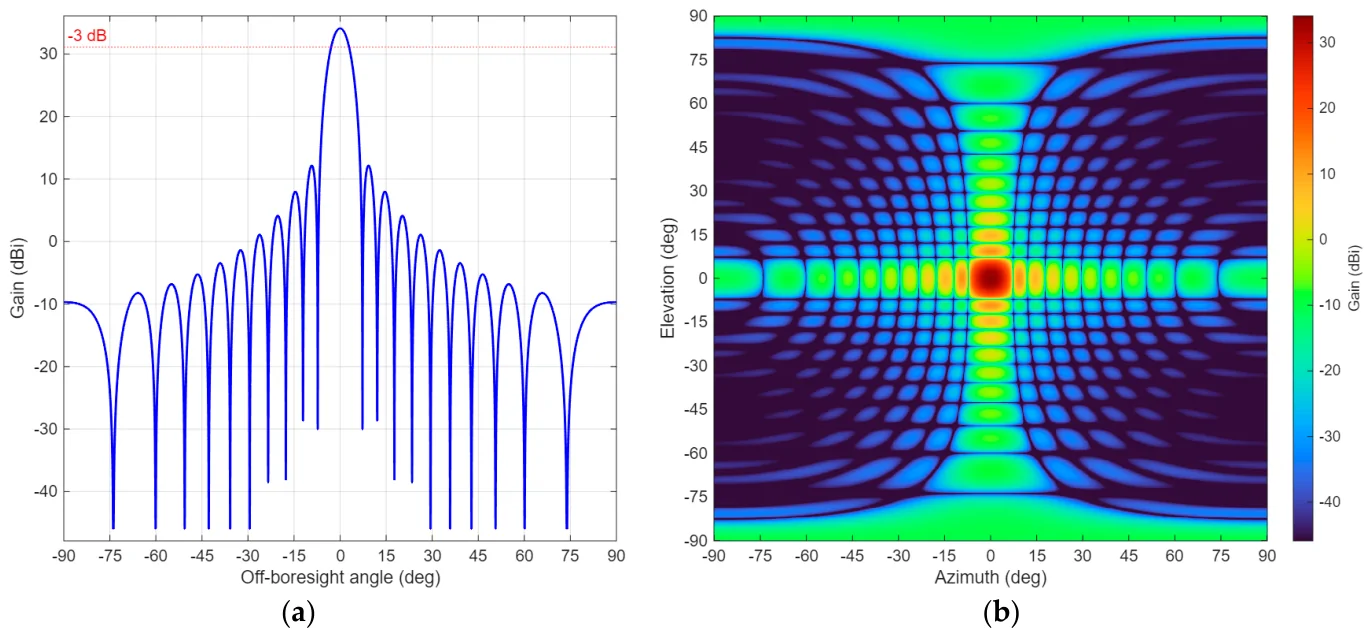
Satellite receive-antenna pattern used in the S-band simulations: (**a**) principal-plane gain versus off-boresight angle; (**b**) gain versus azimuth and elevation. Gain and the numbered color scale are in dBi; angles are in degrees. The specified peak gain is 34.1 dBi (Table 4).

**Figure 3:**
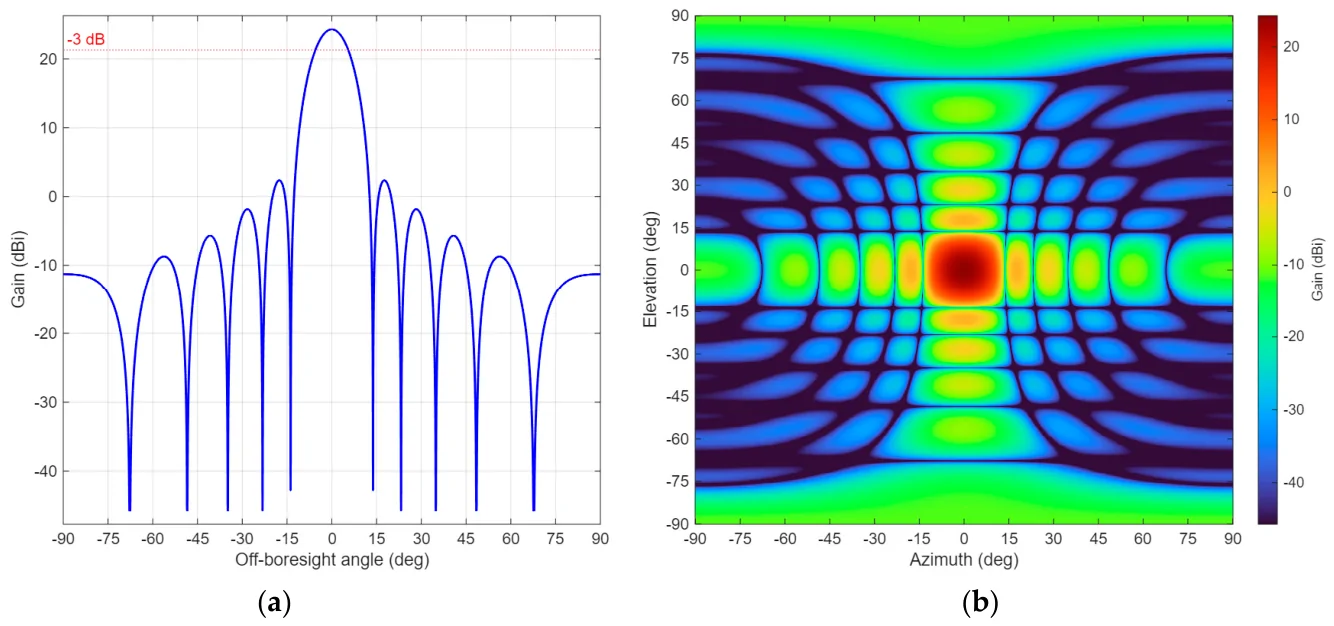
Satellite receive-antenna pattern used in the UHF simulations: (**a**) principal-plane gain versus off-boresight angle; (**b**) gain versus azimuth and elevation. Gain and the numbered color scale are in dBi; angles are in degrees. The specified peak gain is 24.3 dBi (Table 4).

**Figure 4:**
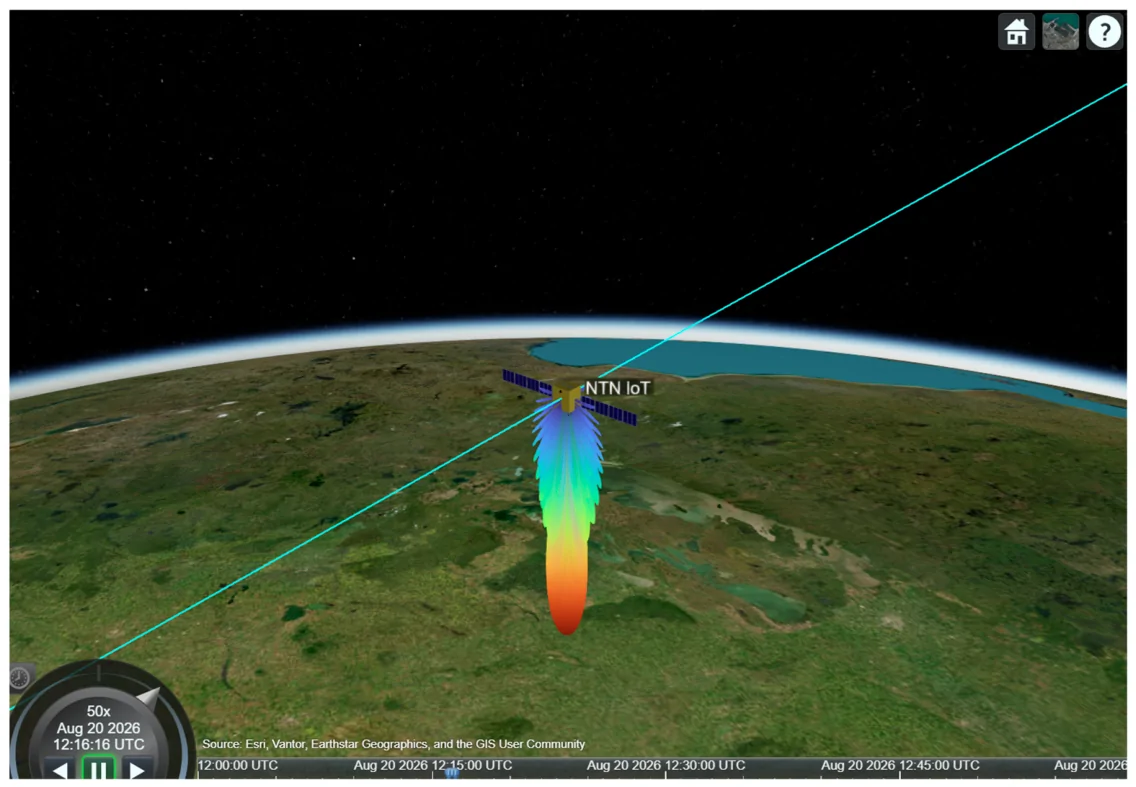
Simulation of the satellite movement.

**Figure 5:**
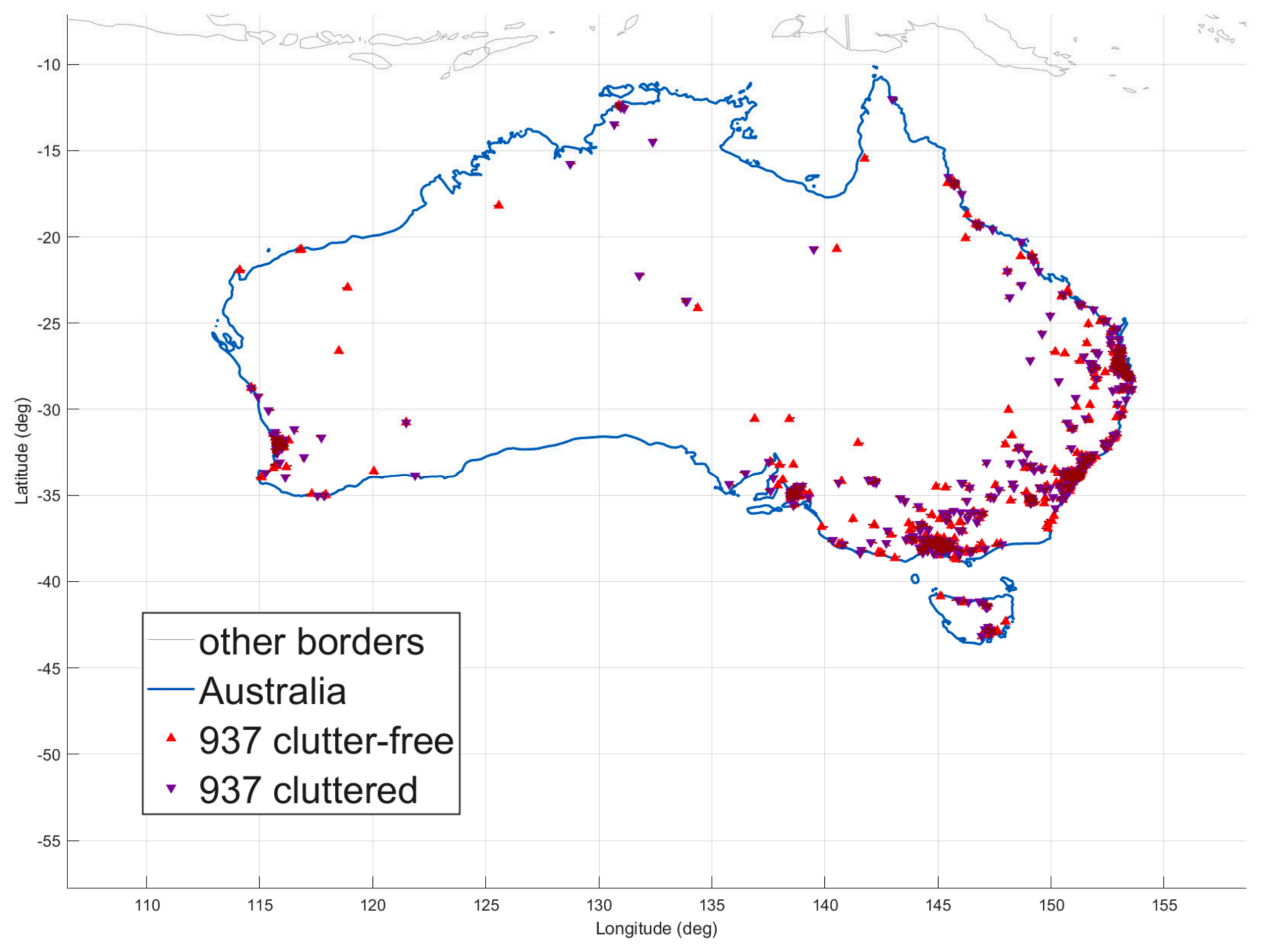
Distribution of BSs in Australia.

**Figure 6:**
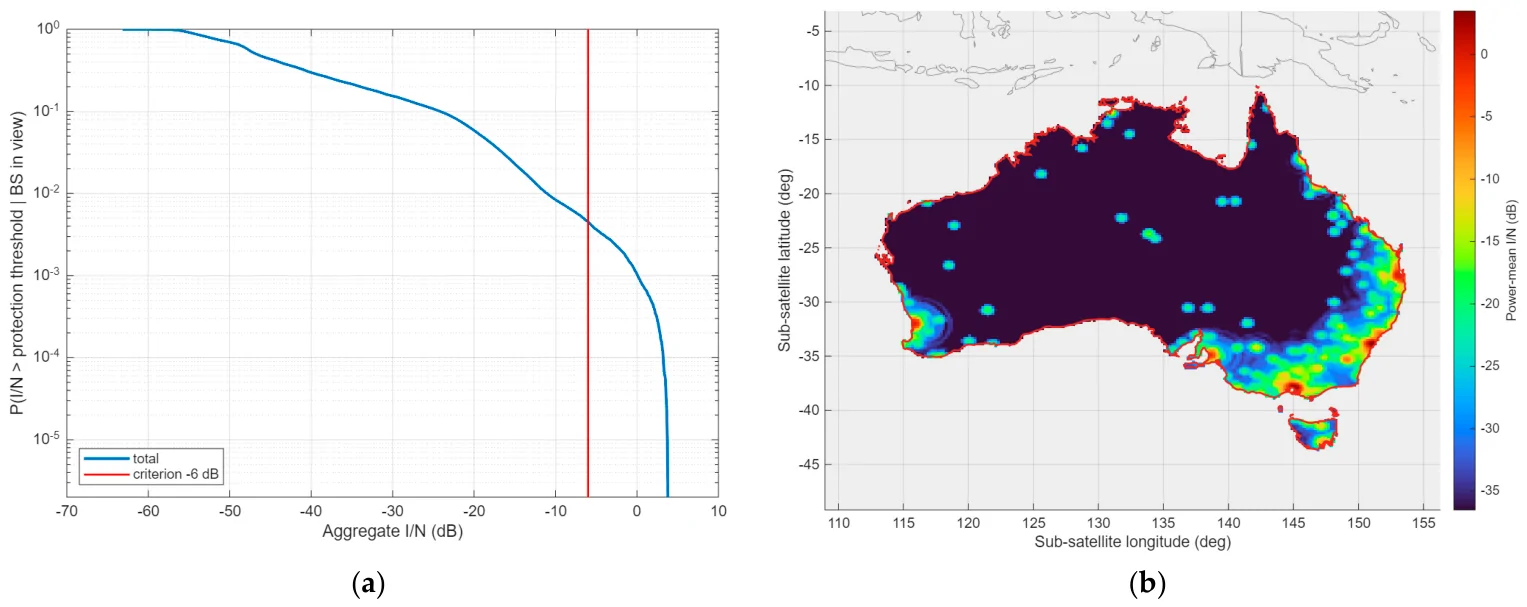
(**a**) CCDF of the I/N levels for nadir pointing of the satellite antenna; (**b**) heatmap of the I/N values over Australia.

**Figure 7:**
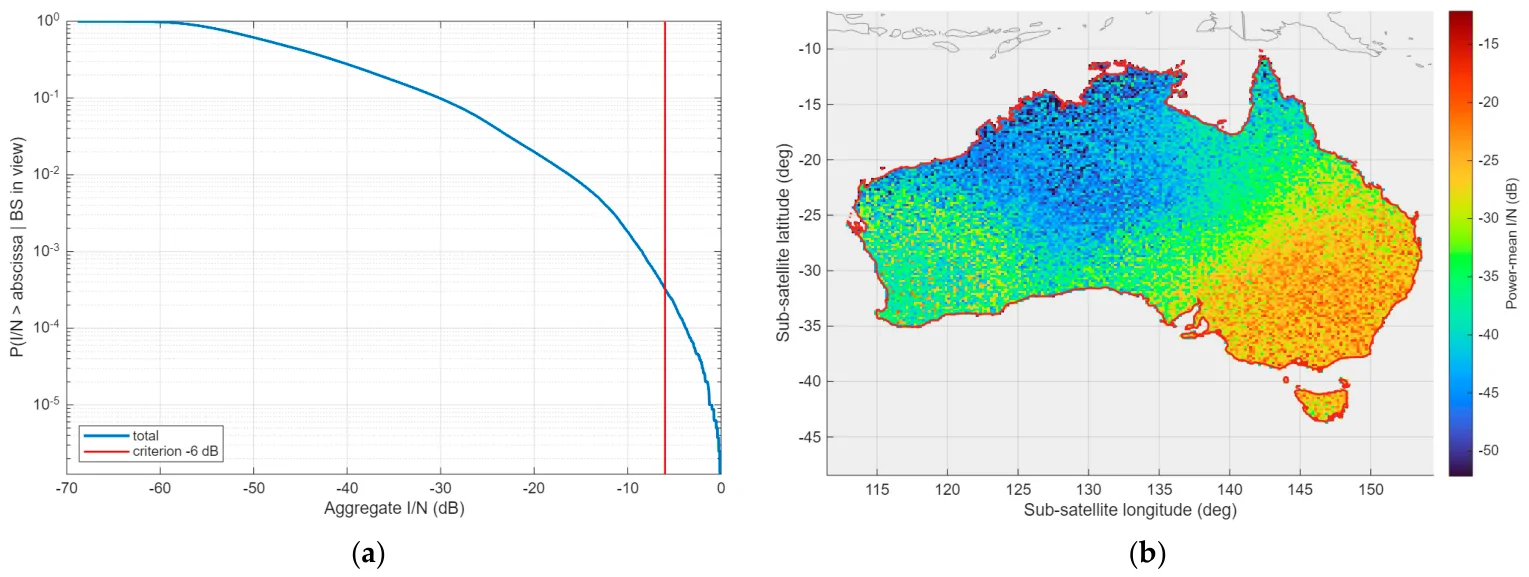
(**a**) CCDF of the I/N levels for off-nadir pointing of the satellite antenna; (**b**) heatmap of the I/N values over Australia.

**Figure 8:**
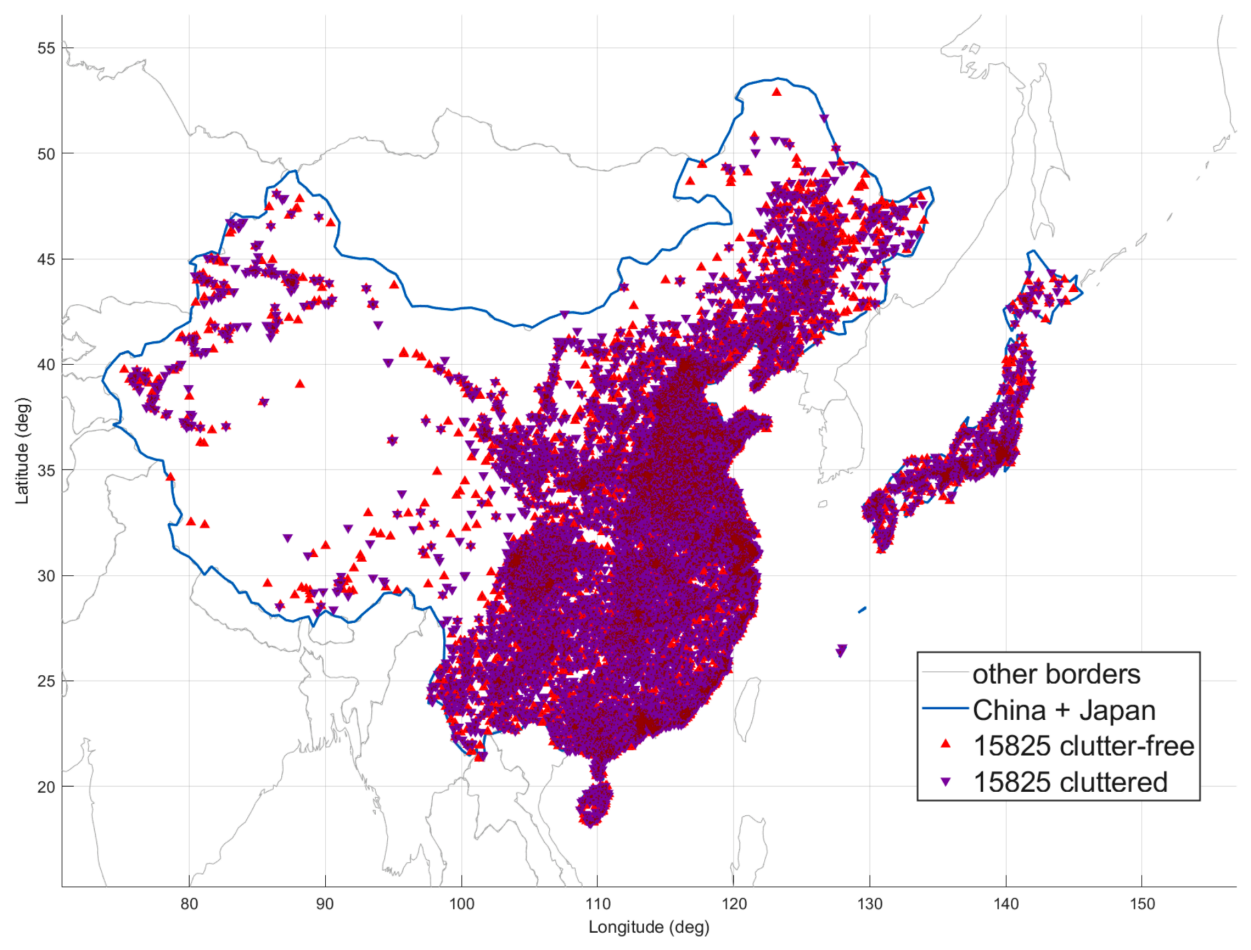
Deployment density of base stations in China and Japan.

**Figure 9:**
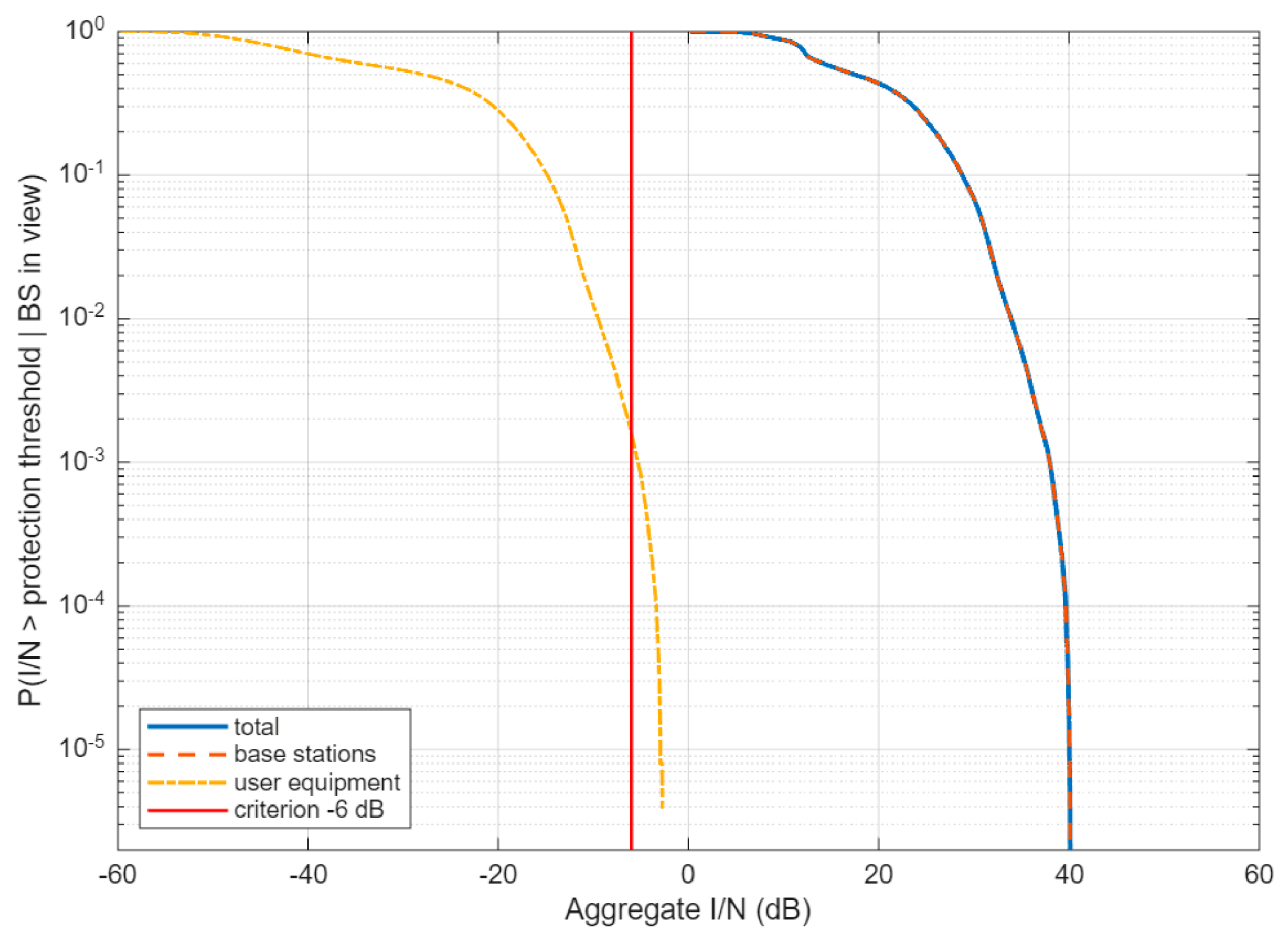
CCDF of the I/N levels inside China and Japan.

**Figure 10:**
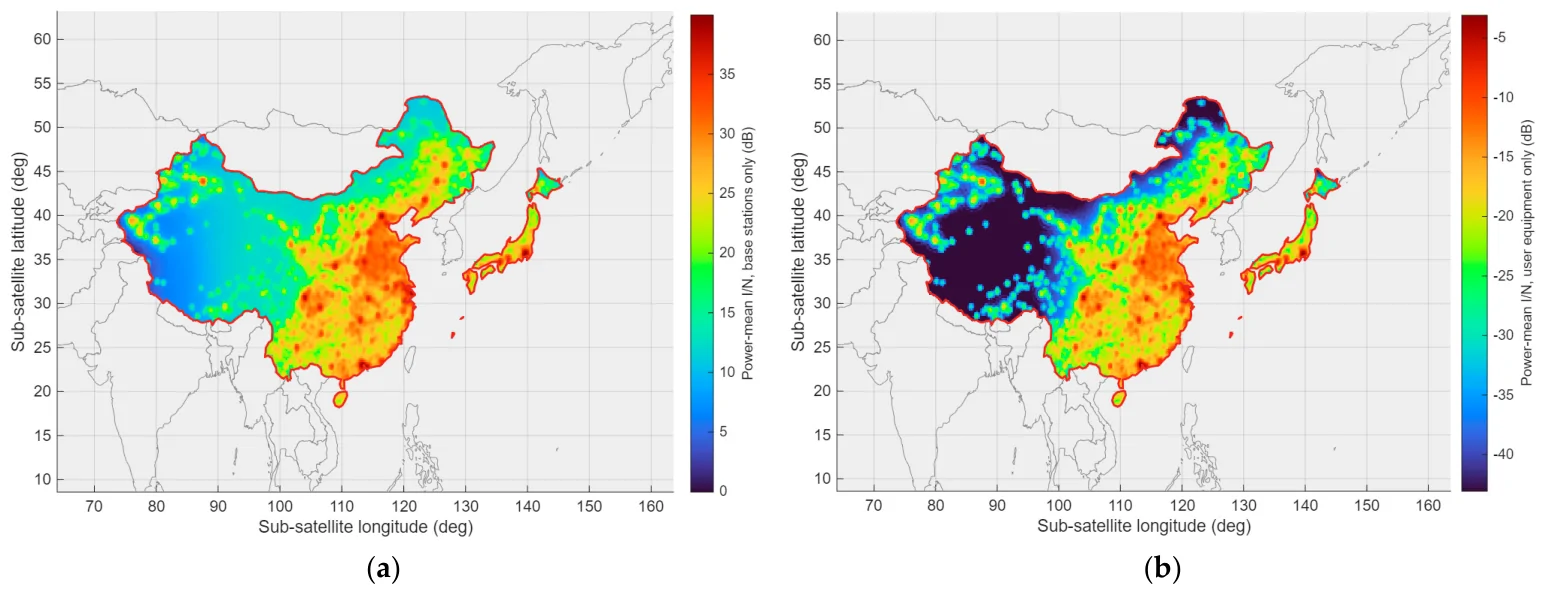
(**a**) Heatmap of the I/N from BSs for nadir pointing of the satellite antenna; (**b**) heatmap of the I/N from UEs over China and Japan.

**Figure 11:**
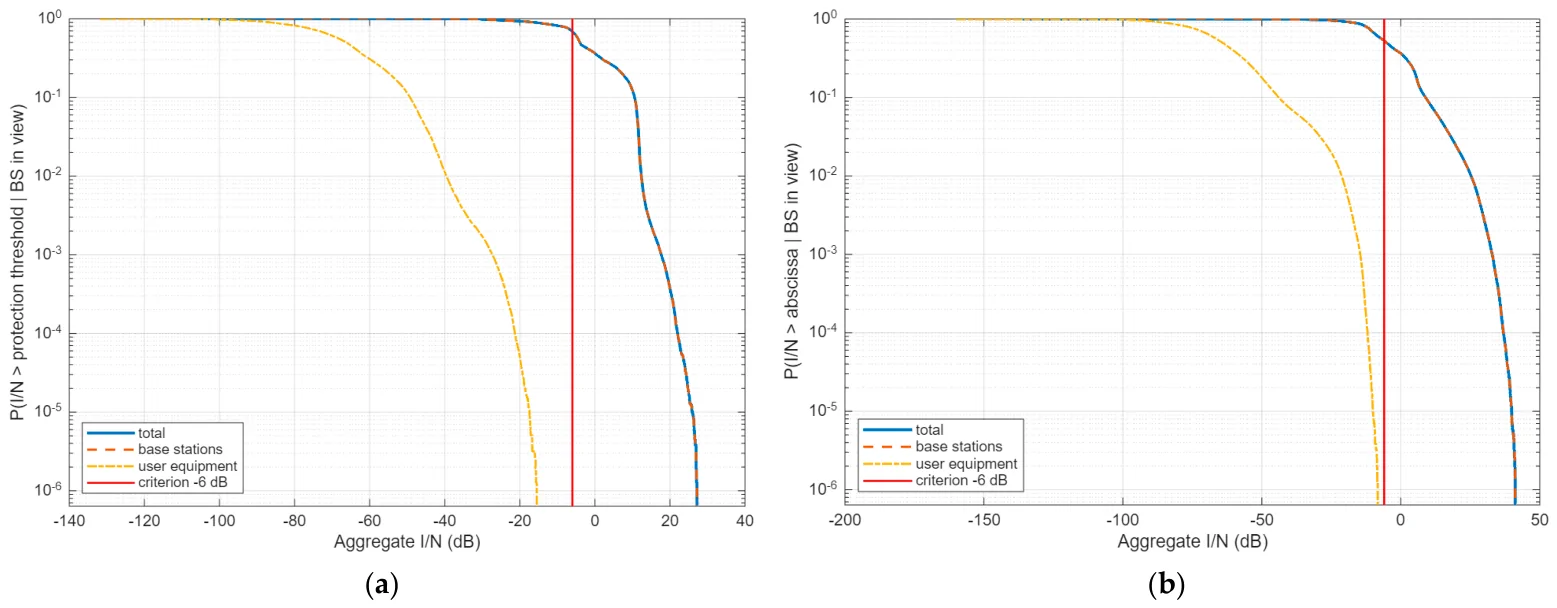
I/N CCDFs for the evaluated domain outside China and Japan: (**a**) nadir pointing; (**b**) random off-nadir pointing. The curves distinguish total, BS, and UE contributions; the red vertical line marks −6 dB. Spatial distributions are shown in Figure 12 and Figure 13.

**Figure 12:**
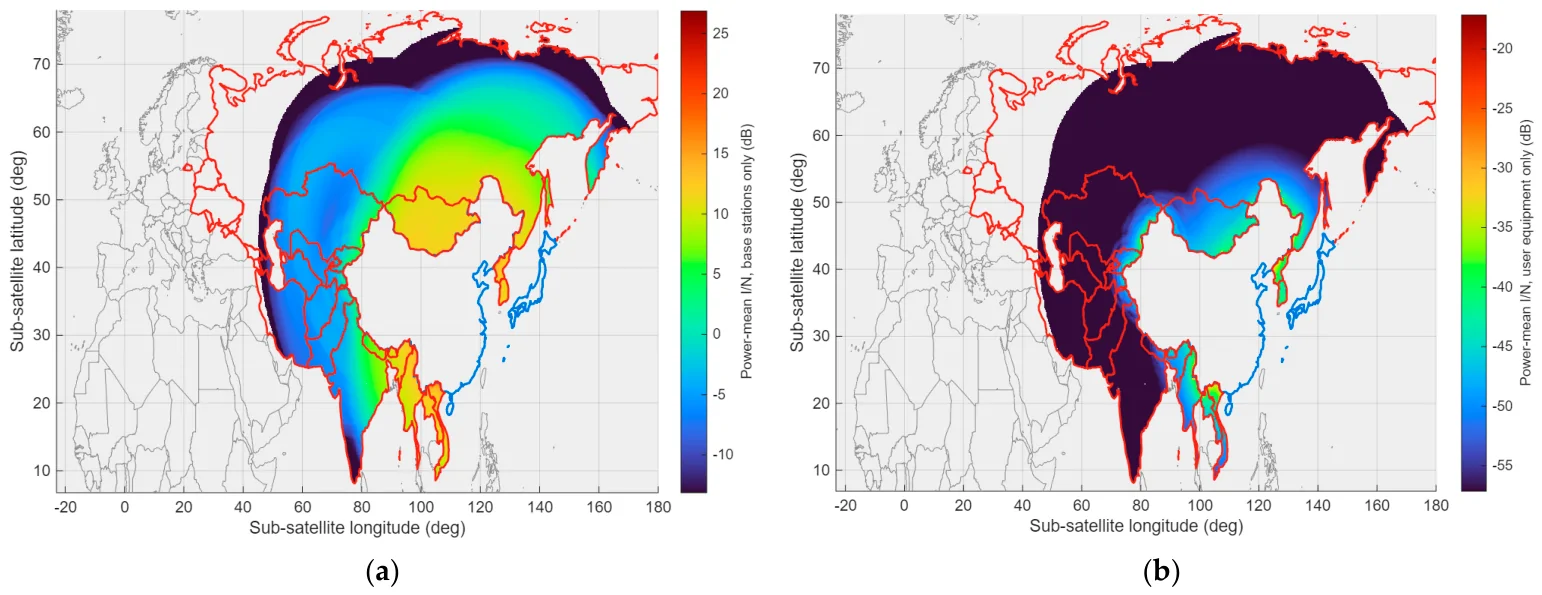
Interference at sub-satellite positions outside China and Japan for nadir pointing: (**a**) BS contribution; (**b**) UE contribution. Color denotes the power-mean I/N in dB, as labeled on each panel. These spatial means are distinct from the sample exceedance probabilities in Figure 11a.

**Figure 13:**
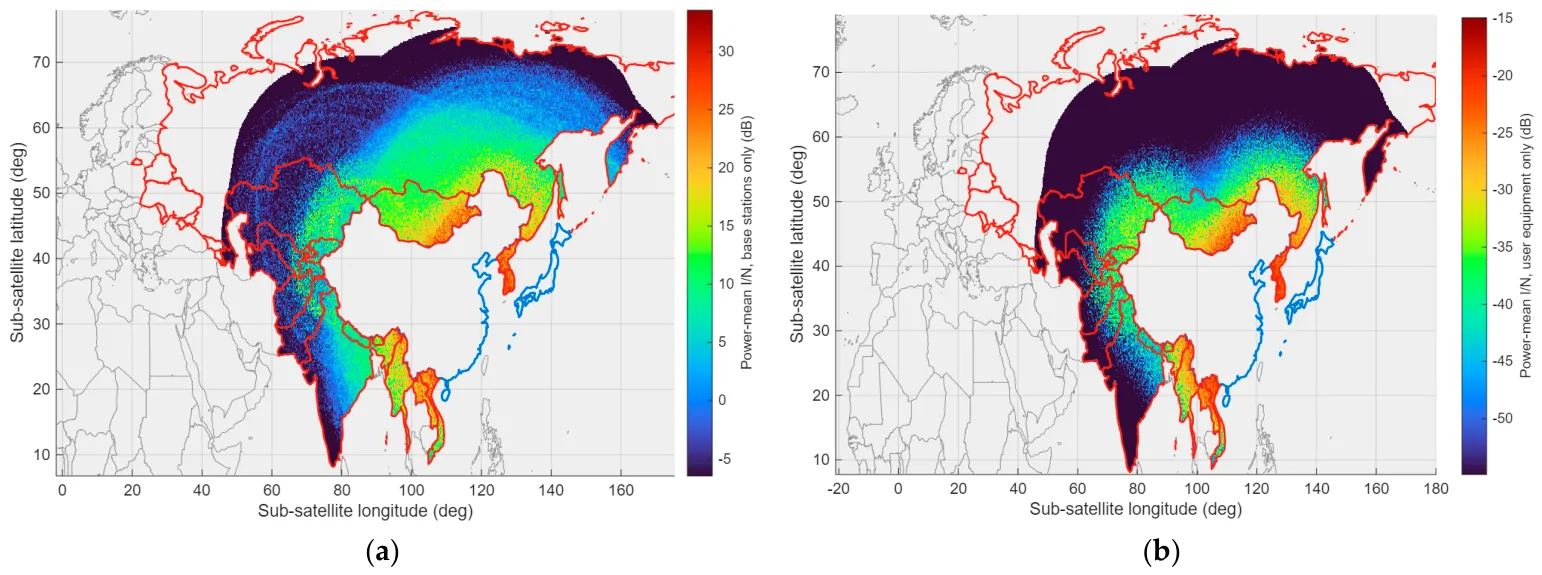
Interference at sub-satellite positions outside China and Japan for random off-nadir pointing: (**a**) BS contribution; (**b**) UE contribution. Color denotes the power-mean I/N in dB, as labeled on each panel. These spatial means are distinct from the sample exceedance probabilities in Figure 11b.

**Figure 14:**
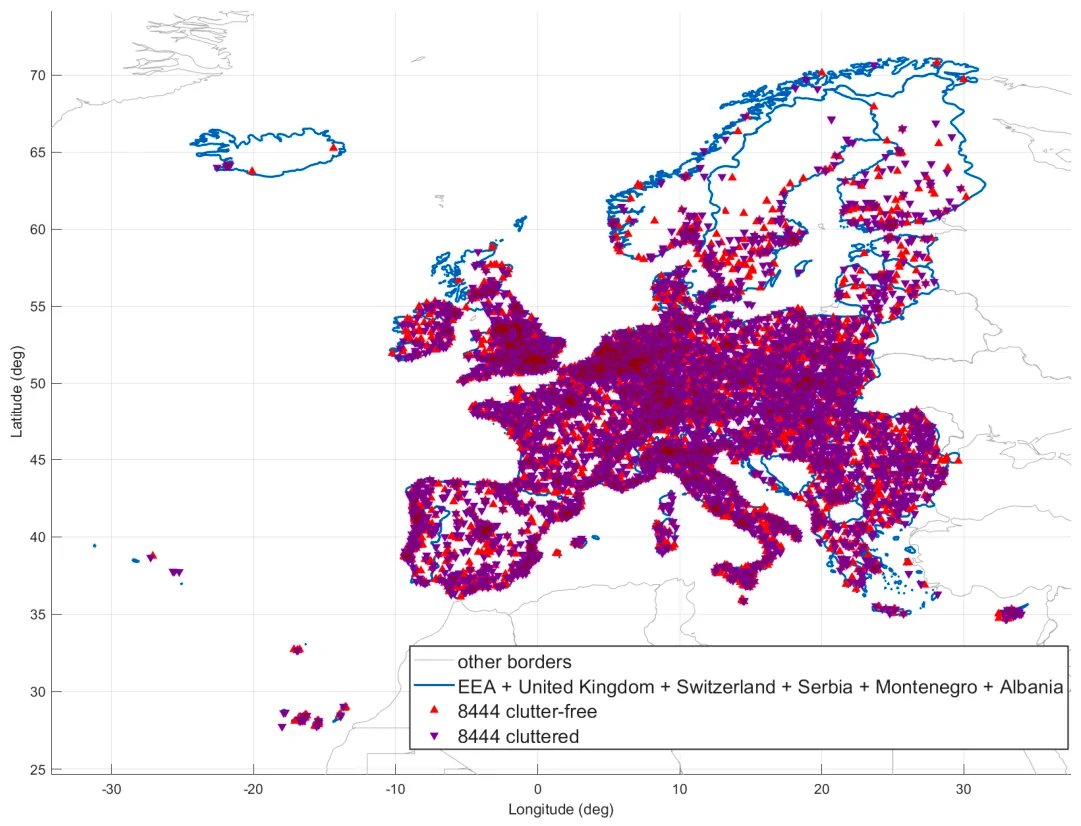
Deployment density of terrestrial networks within the European area.

**Figure 15:**
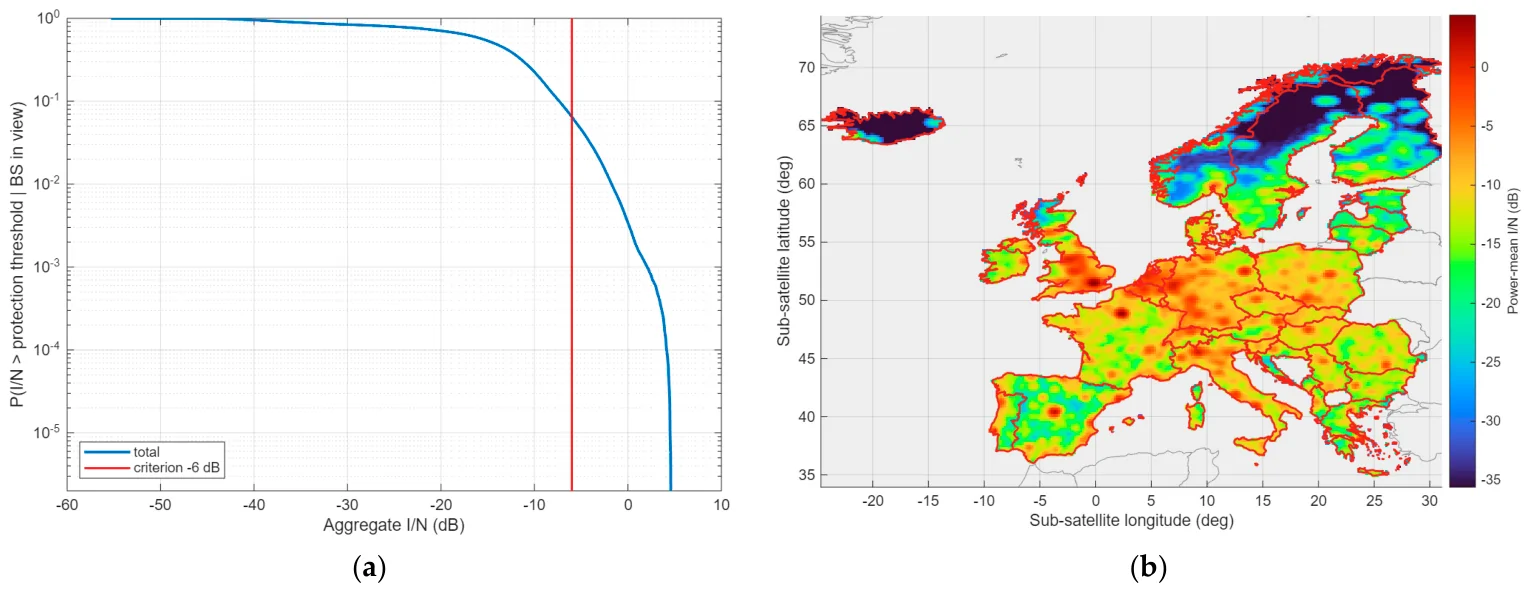
(**a**) CCDF of the I/N levels for nadir pointing of the satellite antenna; (**b**) heatmap of the I/N values over the European area.

**Figure 16:**
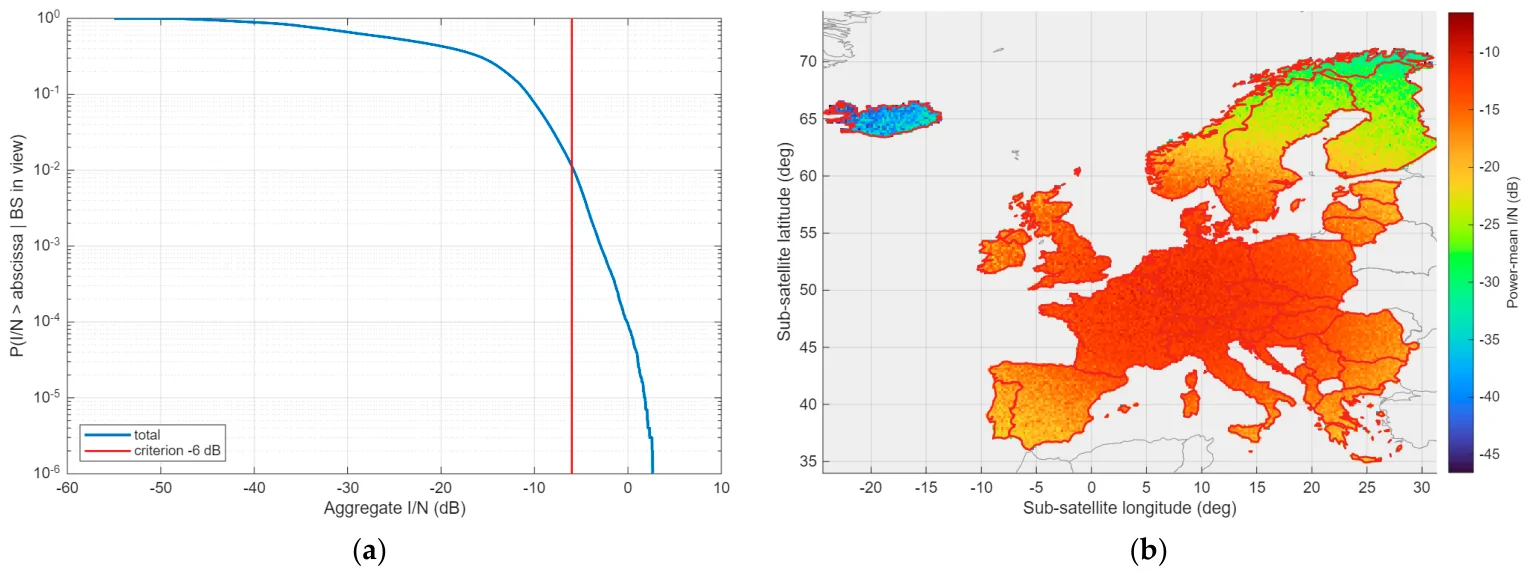
(**a**) CCDF of the I/N levels for off-nadir pointing of the satellite antenna; (**b**) heatmap of the I/N values over the European area.

**Figure 17:**
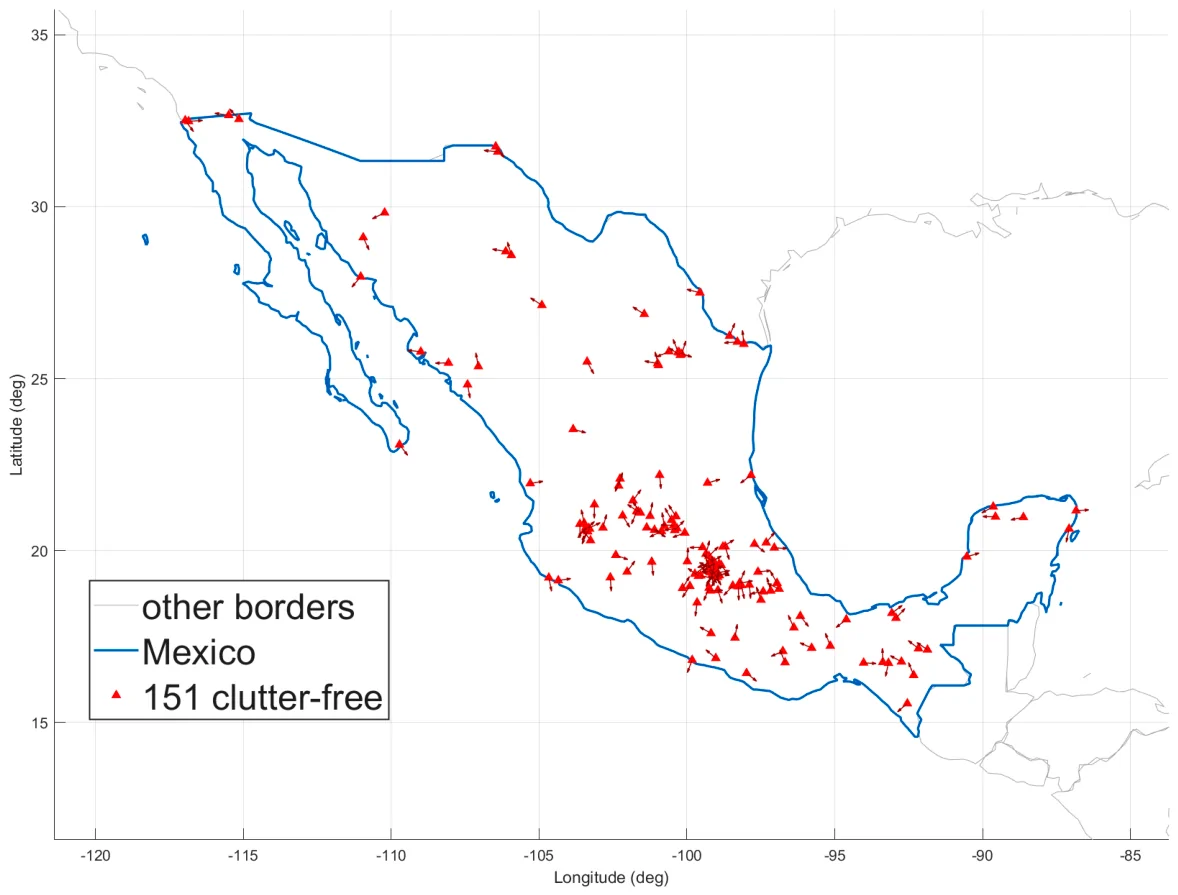
Deployment of base stations in Mexico.

**Figure 18:**
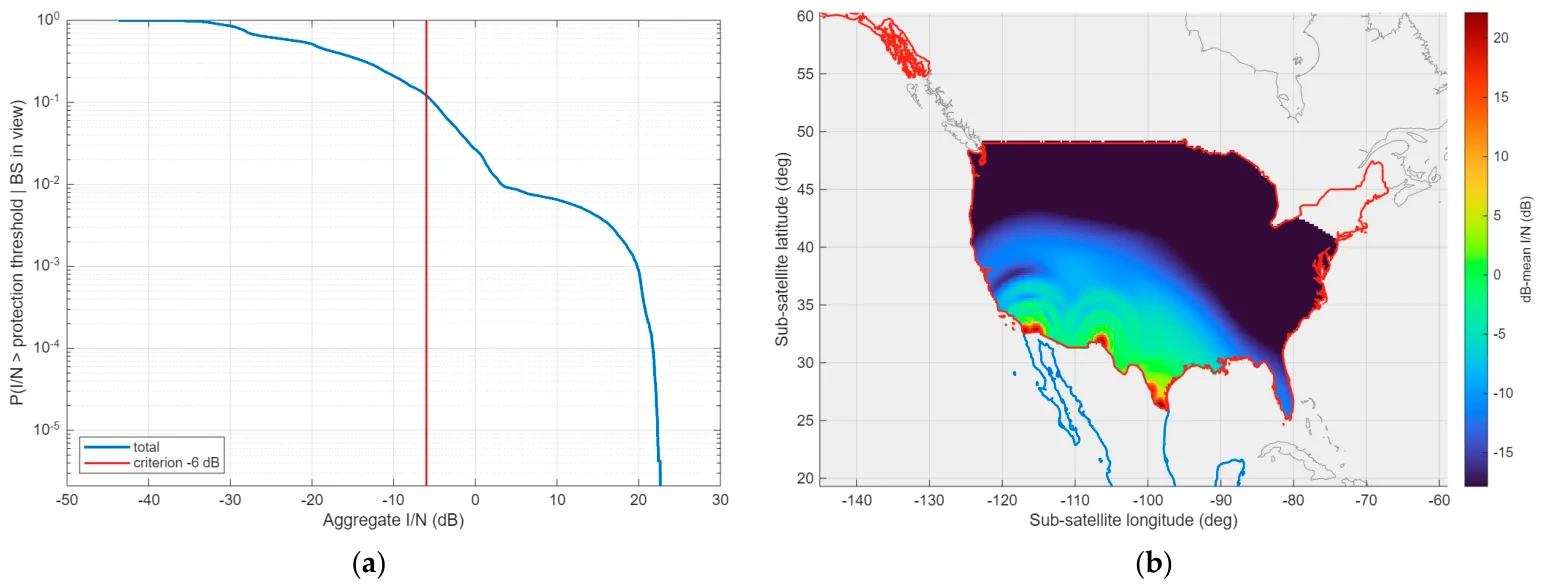
Interference from Mexican BSs for nadir pointing over the United States: (**a**) I/N CCDF, with −6 dB marked in red; (**b**) I/N map at sub-satellite positions over the United States. Panel (**b**) is labeled as a dB-mean map; see the distinction between map statistics and CCDFs in Section 4.

**Figure 19:**
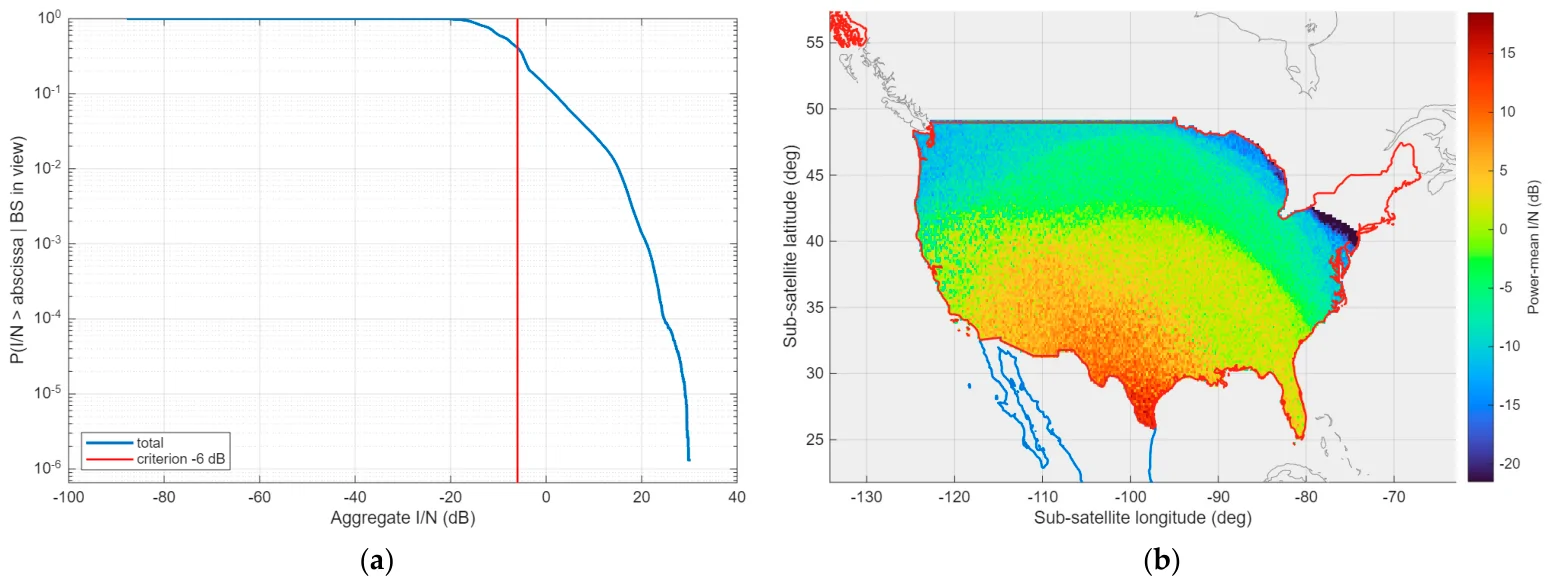
Interference from Mexican BSs for random off-nadir pointing over the United States: (**a**) I/N CCDF, with −6 dB marked in red; (**b**) power-mean I/N map at sub-satellite positions over the United States. The mean convention shown in panel (**b**) differs from the label in Figure 18b; map colors alone are therefore not a controlled measure of the pointing effect.

**Figure 20:**
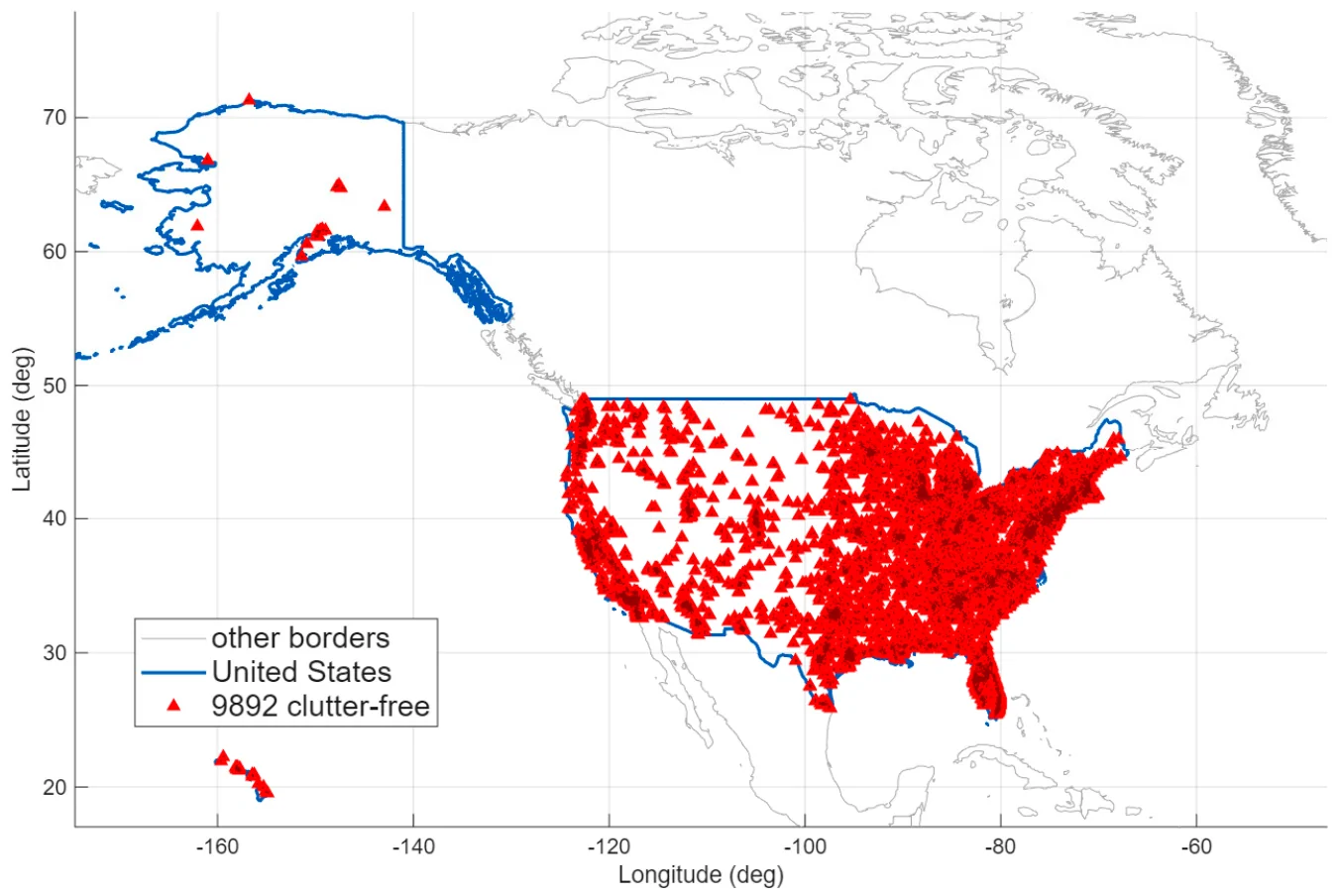
Deployment of base stations in the United States.

**Figure 21:**
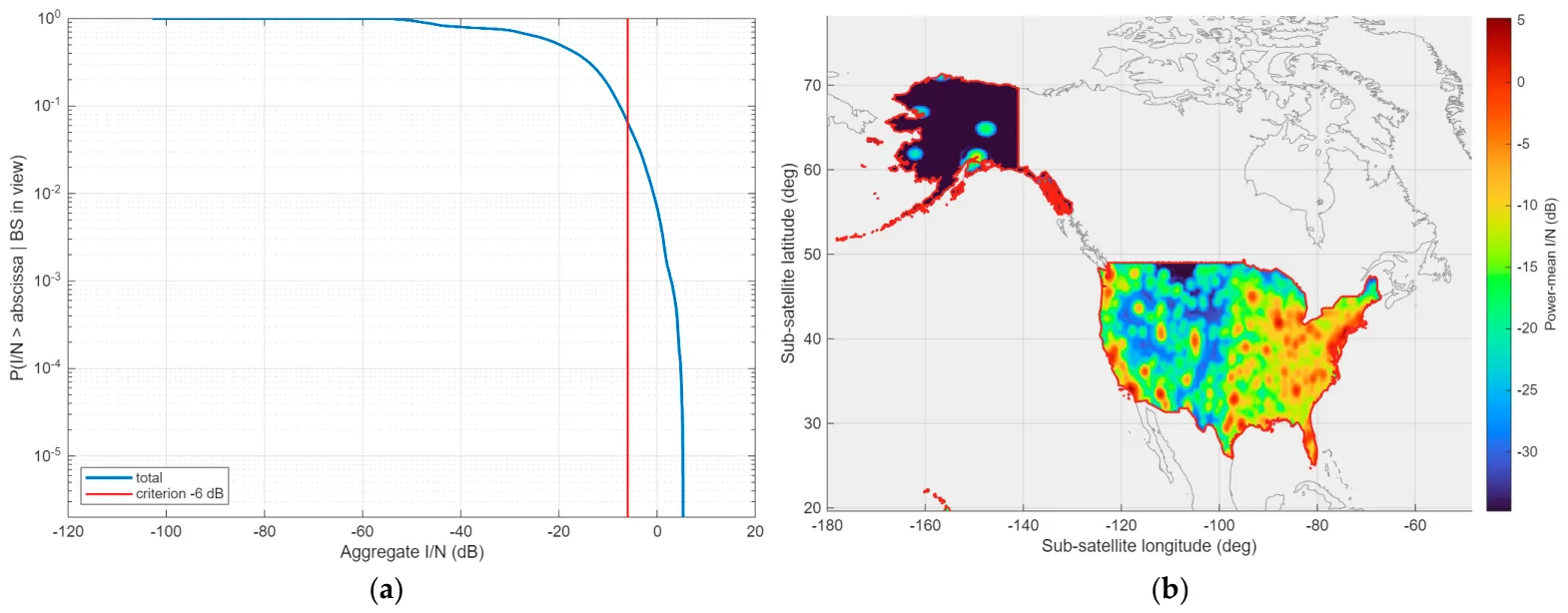
(**a**) CCDF of the I/N levels for nadir pointing of the satellite antenna; (**b**) heatmap of the I/N values in the United States.

**Figure 22:**
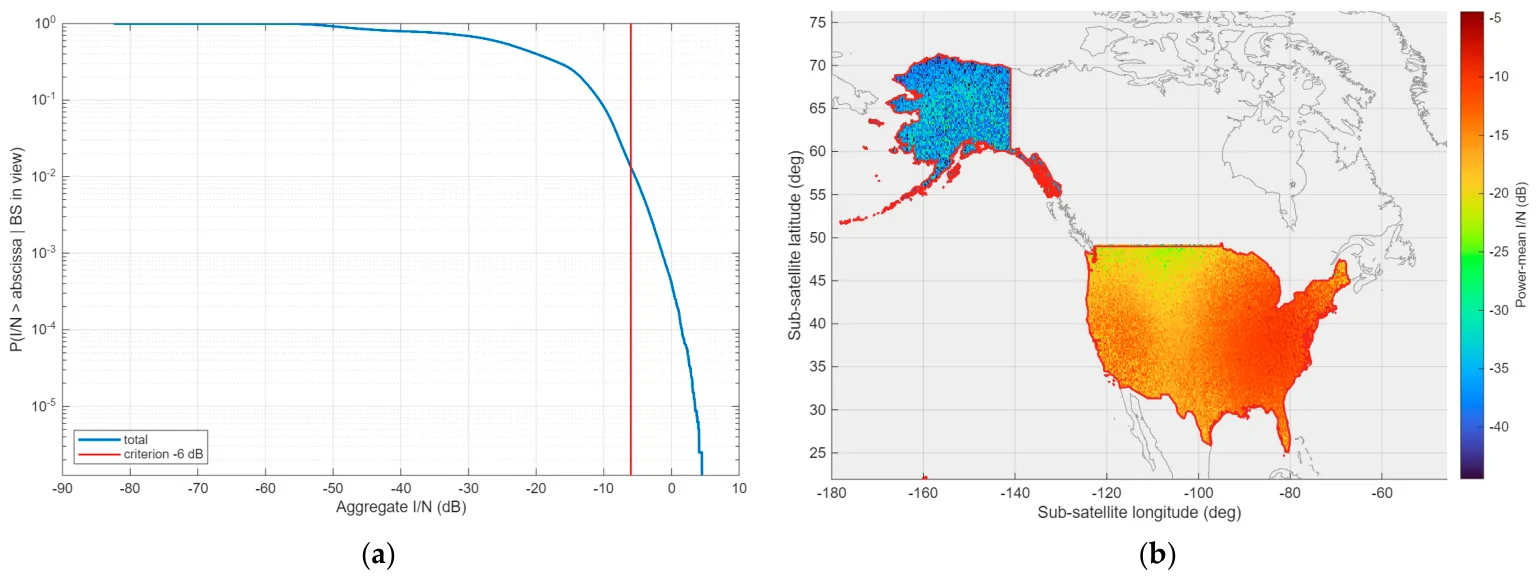
(**a**) CCDF of the I/N levels for off-nadir pointing of the satellite antenna; (**b**) heatmap of the I/N values in the United States.

**Figure 23:**
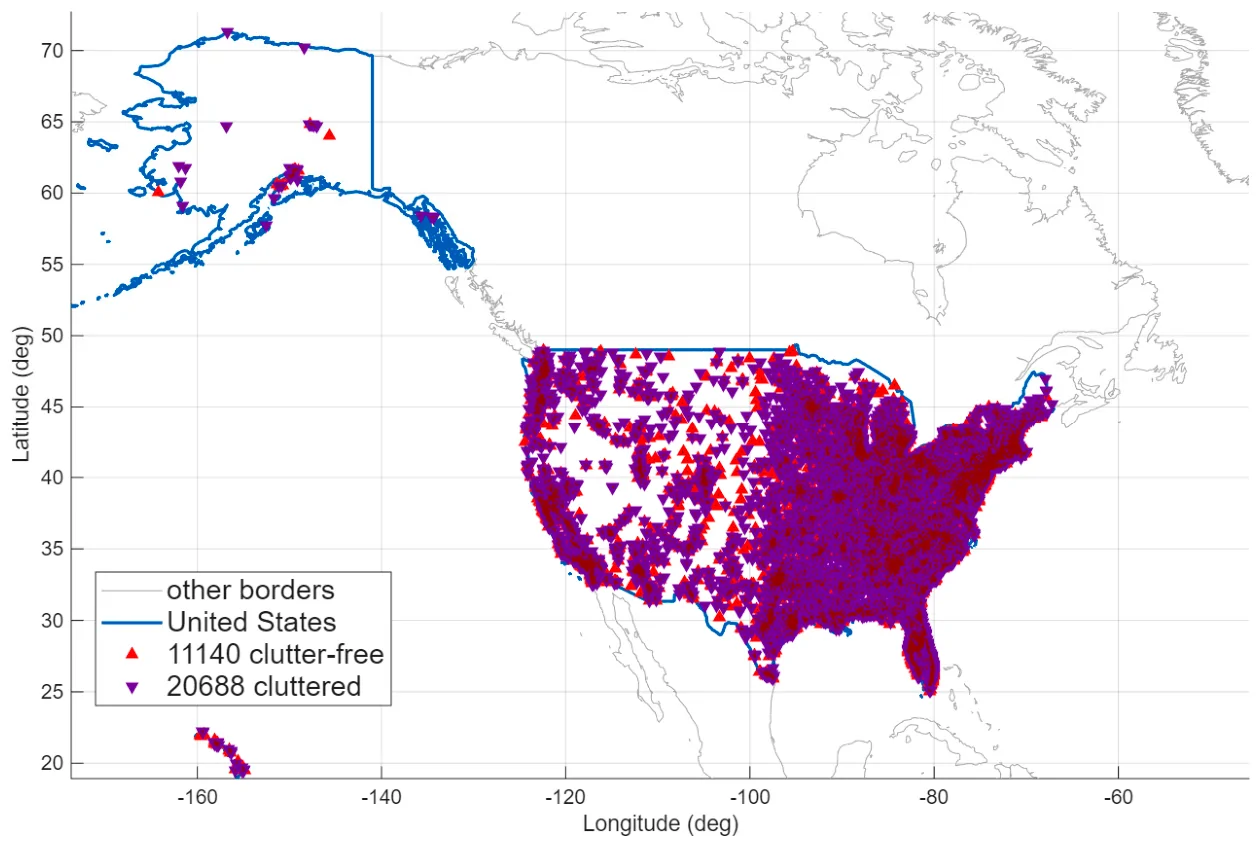
Deployment of base stations within the United States.

**Figure 24:**
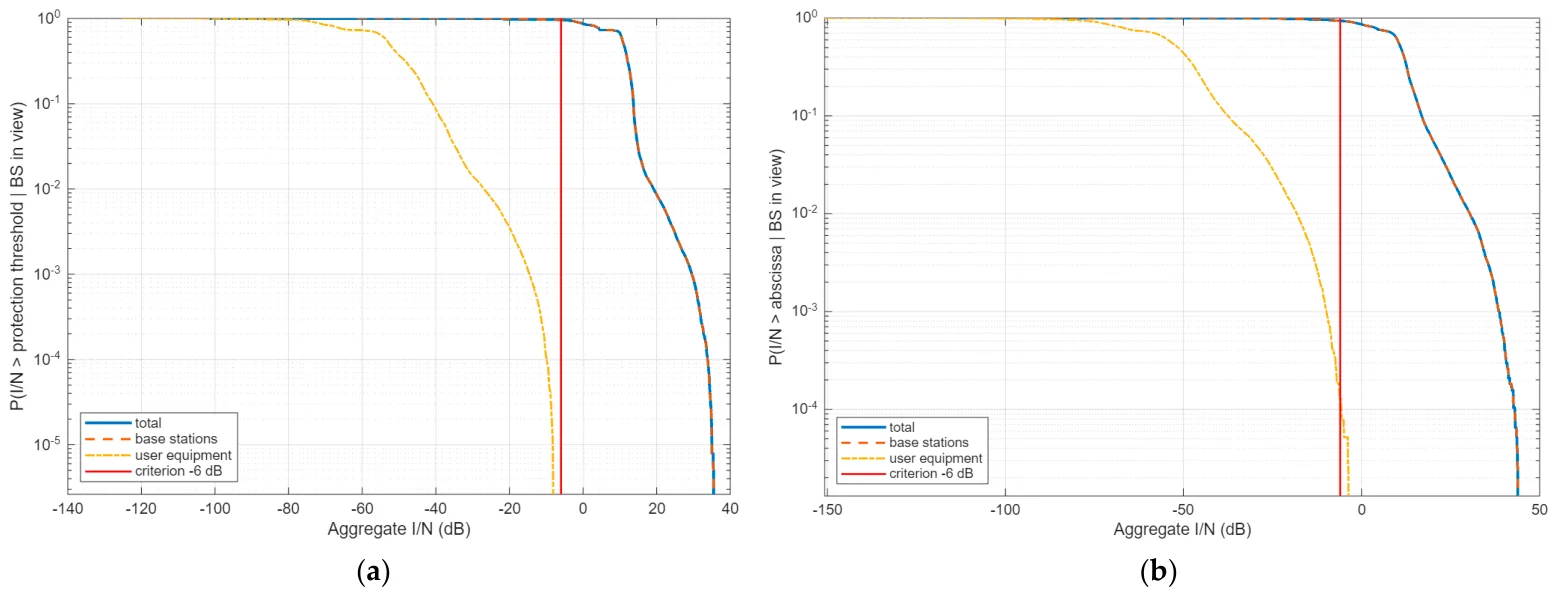
I/N CCDFs for the evaluated domain outside the United States: (**a**) nadir pointing; (**b**) random off-nadir pointing. The curves distinguish total, BS, and UE contributions; the red vertical line marks −6 dB. Spatial distributions are shown in Figure 25 and Figure 26.

**Figure 25:**
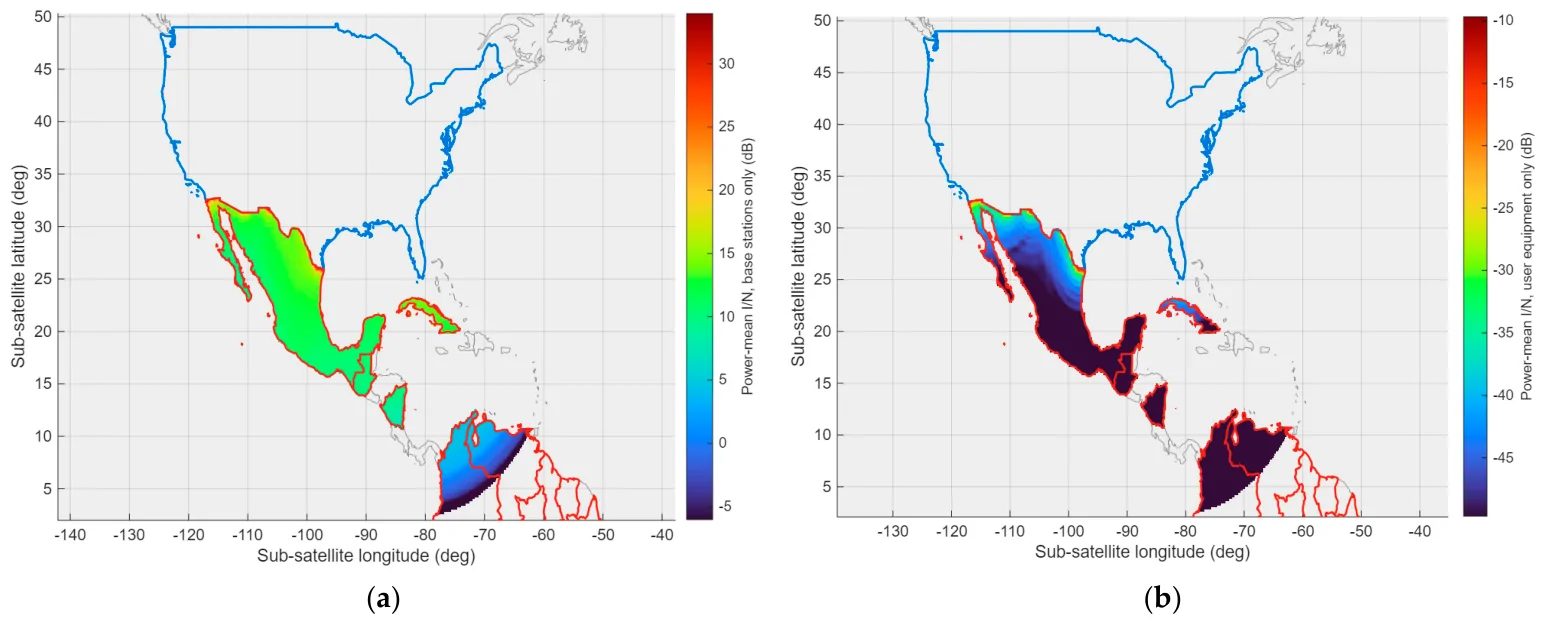
Interference at sub-satellite positions outside the United States for nadir pointing: (**a**) BS contribution; (**b**) UE contribution. The color scale in each panel shows the labeled I/N mean in dB. These spatial means are distinct from the sample exceedance probabilities in Figure 24a.

**Figure 26:**
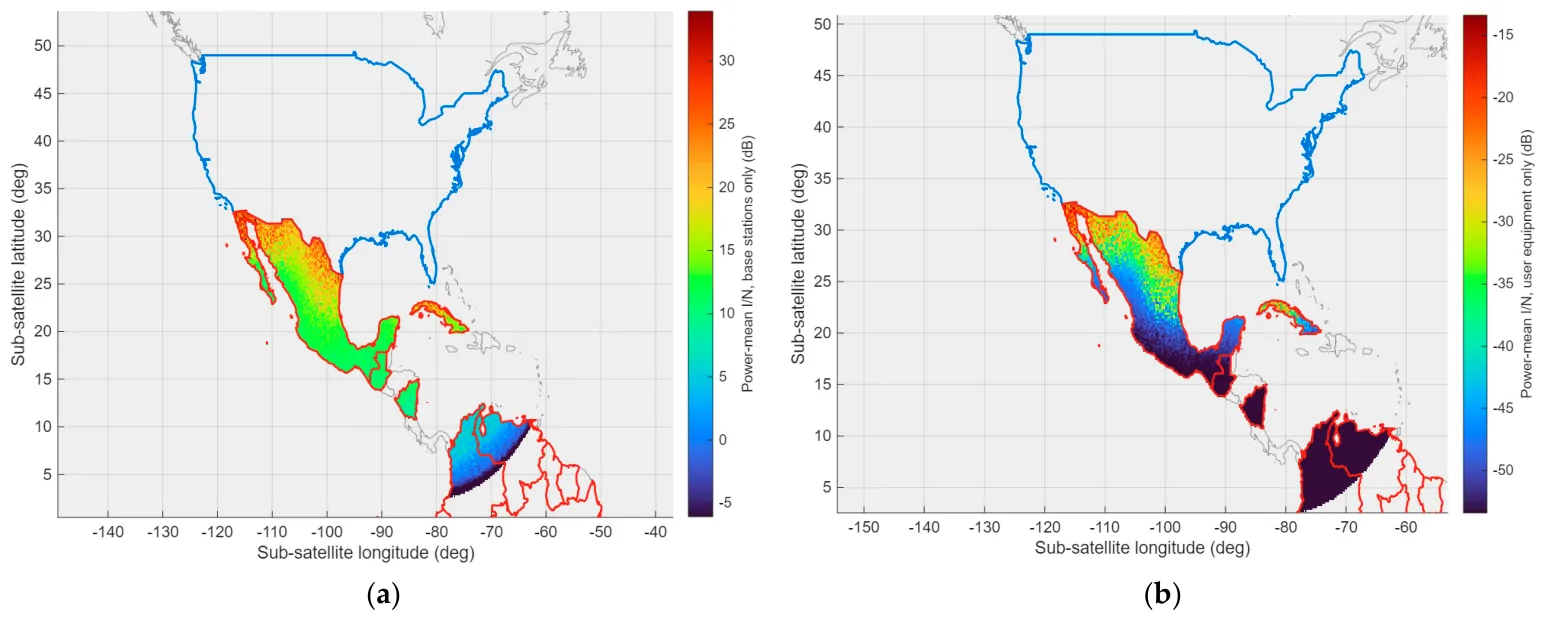
Interference at sub-satellite positions outside the United States for random off-nadir pointing: (**a**) BS contribution; (**b**) UE contribution. The color scale in each panel shows the labeled I/N mean in dB. These spatial means are distinct from the sample exceedance probabilities in Figure 24b.

**Table 1:** Ra and Rb parameters used in the study.

Parameter	Value
Ra	30% Urban (studied area < 200,000 km^2^)
	10% Urban (studied area > 200,000 km^2^)
	10% Suburban (studied area < 200,000 km^2^)
	5% Suburban (area > 200,000 km^2^)
Rb	2.5% (studied area < 200,000 km^2^)
	2% (200,000–1,000,000 km^2^)
	1% (studied area > 1,000,000 km^2^)

**Table 2:** Parameters of the terrestrial networks used in the simulations.

Parameter	UHF	S-Band
Cell radius	>5 km for macro rural scenario 0.5–5 km for macro urban/suburban scenario	0.2–0.8 km for Suburban 0.4–2.5 km for Urban >2 km for Rural
Antenna height	30 m	20–25 m
Sectorization	3 sectors	3 sectors
Downtilt	3 degrees	6–10 degrees
Antenna pattern	Recommendation ITU-R F.1336 (recommends 3.1)	Recommendation ITU-R F.1336 (recommends 3.1)
Below rooftop base station antenna deployment	0%	50%
Maximum base station output power	46 dBm in 10 MHz	46 dBm in 10 MHz
Base station antenna gain	15 dBi	16 dBi
Indoor user terminal usage	50% for macro rural scenario 70% for macro urban/suburban scenario	70%
Average indoor user terminal penetration loss	15 dB for macro rural scenario 20 dB for macro urban/suburban scenario	20 dB
User terminal density in active mode to be used in sharing studies	0.17/5 MHz/km^2^ for macro rural scenario 2.16/5 MHz/km^2^ for urban/suburban scenario	3/5 MHz/km^2^
Maximum user terminal transmitter output power	23 dBm	23 dBm
Average user terminal transmitter output power	2 dBm for macro rural scenario −9 dBm for macro urban/suburban scenario	−9 dBm
Antenna gain for user terminals	−3 dBi	−3 dBi
Body loss	4 dB	4 dB

**Table 3:** Frequency bands where NTN satellites may experience interference from terrestrial networks

NTN Receive Band/ Terrestrial UL (MHz)	Relevant 3GPP Band (s)	Practical Terrestrial Use	Expected Interference
698–716	b12/n12, b17, b85/n85	FDD uplink in North America	UEs only
703–748	b28/n28	FDD uplink in Europe, Asia-Pacific, Latin America and other regions	BS interference possible in 717–748 MHz because of overlapping North American downlinks: b29/n29 SDL at 717–728 MHz, b12/b85 DL at 728/729–746 MHz, and b13 DL above 746 MHz
698–728	b68	FDD uplink in parts of the Middle East and Africa	BS interference possible in 717–728 MHz from b29/n29 SDL where that band is deployed
777–787	b13/n13	FDD uplink, mainly North America	BS interference possible from b28/n28 DL at 758–803 MHz in countries using the APT 700 MHz plan
788–798	b14/n14	FDD public-safety uplink in the United States	BS interference possible from b28/n28 DL and, above 791 MHz, from b20/n20 DL
807–824	b27	FDD uplink; limited regional use	BS interference possible in 807–821 MHz from b20/n20 DL
814–849	b26/n26	FDD uplink, primarily North America and some Asian markets	BS interference possible in 814–821 MHz from b20/n20 DL; otherwise UEs only
815–830	b18/n18	FDD uplink in Japan	BS interference possible in 815–821 MHz from b20/n20 DL; otherwise UEs only
824–849	b5/n5	Widely used FDD uplink	UEs only
830–845	b19	FDD uplink in Japan	UEs only
832–862	b20/n20	FDD uplink in Europe and other Region 1 countries	Mainly UEs only; BS interference is possible in the upper part, particularly 859–862 MHz, from b26/n26 DL. If b27 is present, the overlap may begin at 852 MHz
880–915	b8/n8	Widely used FDD uplink	BS interference possible in 880–894 MHz from b5/n5, b18, b19 and b26/n26 downlinks; 894–915 MHz is UE-only
1427–1470	b11, b21, b74/n74	FDD uplink arrangements; b11/b21 are used in Japan, while b74 is less widespread	BS interference possible, particularly in 1452–1470 MHz, from European b32 SDL; wider overlap is possible where n75/n76 SDL is deployed
1710–1785	b3/n3	Widely used FDD uplink	UEs only
1710–1780	b66/n66	FDD uplink, principally North America	UEs only
1850–1910/1915	b2/n2, b25/n25	FDD uplink, principally North America	BS interference possible in 1850–1880 MHz from b3/n3 DL and in 1880–1915 MHz from b39/n39 TDD deployments
1920–1980	b1/n1	Widely used FDD uplink	BS interference possible in 1930–1980 MHz from b2/n2 and b25/n25 downlinks; 1920–1930 MHz is normally UE-only
1880–1920	b39/n39	TDD, particularly in China	BSs and UEs
2010–2025	b34/n34	Regional TDD use, primarily in China	BSs and UEs
2305–2315	b30/n30	FDD uplink in the United States	BS interference possible from b40/n40 TDD networks operating at 2300–2400 MHz in other regions
2300–2400	b40/n40	Commercial TDD use in several Asian and European markets	BSs and UEs
2500–2570	b7/n7	Widely used FDD uplink	BS interference possible from b41/n41 TDD networks operating at 2496–2690 MHz
2570–2620	b38/n38	Commercial TDD use	BSs and UEs
2496–2690	b41/n41	Extensive commercial TDD use, including the United States and Asian markets	BSs and UEs

**Table 4:** Characteristics of the NTN satellite systems used in simulations.

Parameter	UHF	S-Band
Orbit height	500	340/525
Inclination	53	53
Noise bandwidth	180 kHz	180 kHz
Antenna pattern	Using 1.4 of ITU-R S.1528, use the following:	Using 1.4 of ITU-R S.1528, use the following:
	G_max_ = 24.3 dBi,	G_max_ = 34.1 dBi,
	L_r_ = L_t_ = 2.0 m,	L_r_ = L_t_ = 1.6 m,
	SLR = 20 dB,	SLR = 20 dB,
	l = 2,	l = 2,
	λ = 0.35 m.	λ = 0.15 m.
Noise temperature	420 K	778 K
Protection threshold	I/N = −6 dB	I/N = −6 dB

**Table 5:** Satellite receiver’s antenna parameters required for forming the antenna pattern.

Symbol	Parameter	Unit
G_max_	Peak gain	dBi
SLR	Side-lobe ratio	dB
*l*	Number of secondary lobes retained in the Taylor product	-
λ	Wavelength	m
Lr, Lt	Radial and transverse dimensions of the effective radiating area	m
α, ε	Azimuth and elevation in the antenna fixed frame	deg
θ	Off-boresight angle	deg
φ	Roll angle about the boresight axis	deg
$n_{x},\;n_{y},\;n_{z}$	Unit direction vector in the antenna frame	-
$J_{1}$	Bessel function of the first kind, order 1	-
$j_{l,\;i}$	*i*-th positive zero of $J_{1}$	-

## Data Availability

The original contributions presented in this study are included in the article. Further inquiries can be directed to the corresponding authors.

## Abbreviations

The following abbreviations are used in this manuscript:

BS	Base station
CCDF	Complementary cumulative distribution function
D2C	Direct-to-cell
DL	Downlink
FDD	Frequency-division duplexing
IMT	International Mobile Telecommunications
IoT	Internet of Things
ITU-R	International Telecommunication Union Radiocommunication Sector
LEO	Low-Earth orbit
MSS	Mobile-satellite service
NTN	Non-terrestrial network
TDD	Time-division duplexing
UE	User equipment
UL	Uplink
WRC	World Radiocommunication Conference
